# Tumoral microenvironment prevents de novo asparagine biosynthesis in B cell lymphoma, regardless of ASNS expression

**DOI:** 10.1126/sciadv.abn6491

**Published:** 2022-07-06

**Authors:** Manuel Grima-Reyes, Ashaina Vandenberghe, Ivan Nemazanyy, Pauline Meola, Rachel Paul, Julie Reverso-Meinietti, Adriana Martinez-Turtos, Nicolas Nottet, Wai-Kin Chan, Philip L. Lorenzi, Sandrine Marchetti, Jean-Ehrland Ricci, Johanna Chiche

**Affiliations:** 1 Université Côte d’Azur, Inserm, C3M, Nice, France.; 2 Equipe labellisée Ligue Contre le Cancer, Nice, France.; 3 Plateforme d’étude du métabolisme SFR-Necker, Inserm US 24–CNRS UAR, 3633 Paris, France.; 4 Department of Bioinformatics and Computational Biology, The University of Texas MD Anderson Cancer Center, Houston, TX, USA.

## Abstract

Depletion of circulating asparagine with l-asparaginase (ASNase) is a mainstay of leukemia treatment and is under investigation in many cancers. Expression levels of asparagine synthetase (ASNS), which catalyzes asparagine synthesis, were considered predictive of cancer cell sensitivity to ASNase treatment, a notion recently challenged. Using [U-^13^C_5_]-l-glutamine in vitro and in vivo in a mouse model of B cell lymphomas (BCLs), we demonstrated that supraphysiological or physiological concentrations of asparagine prevent de novo asparagine biosynthesis, regardless of ASNS expression levels. Overexpressing ASNS in ASNase-sensitive BCL was insufficient to confer resistance to ASNase treatment in vivo. Moreover, we showed that ASNase’s glutaminase activity enables its maximal anticancer effect. Together, our results indicate that baseline ASNS expression (low or high) cannot dictate BCL dependence on de novo asparagine biosynthesis and predict BCL sensitivity to dual ASNase activity. Thus, except for ASNS-deficient cancer cells, ASNase’s glutaminase activity should be considered in the clinic.

## Teaser

ASNS is insufficient to determine lymphoma dependence on de novo asparagine synthesis and to confer resistance to l-asparaginase.

## INTRODUCTION

Cancer cells rewire their metabolism to fulfill the energetic and biosynthetic demands of tumor growth. Beyond their widely known avidity for glucose, tumors are also addicted to nonessential amino acids (NEAAs) despite their ability to synthetize them. This can be explained in part by insufficient production of intracellular NEAAs relative to tumor needs and/or by epigenetic modifications leading to silencing of genes encoding metabolic enzymes involved in the synthesis of specific NEAAs. Accordingly, some NEAAs were reclassified as semi-essential amino acids (SEAAs) in the context of cancer. Unlike other amino acids (AAs), the NEAA asparagine is not catabolized by mammalian cells (*1*) and is known to play a key role in tumor progression. Asparagine is sufficient to reduce glutamine depletion–induced apoptosis in *Myc*-transformed cells in vitro (*2*) and is critical for cell survival and growth in vivo as tumors become depleted of glutamine (*1*). Recently, asparagine was found to be limiting for cancer cell survival and proliferation upon electron transport chain inhibition (*3*). Moreover, in a mouse model of breast cancer, asparagine availability has been evidenced as a key determinant of metastasis formation (*4*). Thus, many successful efforts have been made to discover the metabolic functions of asparagine in cancer cells, with the aim of showing that it is not exclusively used as a building block for protein synthesis. To favor cancer cell anabolism and proliferation, asparagine coordinates protein and nucleotide synthesis by signaling to mammalian target of rapamycin complex 1 (mTORC1) (*5*). To do so, intracellular asparagine is exchanged with specific extracellular AAs, including arginine and serine, which contribute to mammalian target of rapamycin complex 1 (mTORC1) activation and nucleotide biosynthesis, respectively (*5*). Asparagine was also described to regulate mTORC1 activity through Arf1 in the absence of the Rag–GTPases (guanosine triphosphatases) (*6*).

Asparagine has long been accepted as a metabolite to be targeted in cancer. The first evidence was shown by treating lymphoma-bearing mice with guinea pig serum, which resulted in tumor regression (*7*). Later, the presence of l-asparaginase (ASNase) in this serum was demonstrated to be responsible for this anticancer effect (*8*). ASNase induces the depletion of circulating asparagine by catalyzing its hydrolysis, thus generating aspartate (Asp) and the toxic by-product ammonium. ASNase is also able to reduce circulating glutamine levels because of its glutaminase activity, but its affinity for glutamine is much lower than for asparagine. Since 1978, ASNase has been the only therapeutic enzyme targeting tumor addiction to specific circulating SEAAs that is U.S. Food and Drug Administration approved for its clinical use in chemotherapeutic regimens to treat childhood and adult acute lymphoblastic leukemias (ALLs). The rationale behind its unique recommendation in ALL is that most leukemic lymphoid cells display an epigenetic silencing of the gene encoding the enzyme asparagine synthetase (ASNS), which catalyzes the adenosine 5′-triphosphate (ATP)–dependent conversion of aspartate and glutamine into asparagine and glutamate. Consequently, ALL cells are unable to synthesize asparagine, and their survival and division rely on the availability of microenvironmental sources (asparagine auxotrophy), which makes them sensitive to ASNase therapy. Currently, ASNase is under clinical investigation in a large panel of hematopoietic and solid cancers. Unfortunately, ASNS silencing is rare in tumors other than ALLs, and it remains a major challenge to predict ASNS-expressing cells’ sensitivity to ASNase therapy.

Given the importance of asparagine in cancer cell proliferation, ASNS upregulation has long been associated with ASNase resistance. Such a claim would indicate that the level of ASNS expression is directly correlated with its activity, i.e., the level of asparagine synthetized by the cell. Few attempts have been made to prove this in in vitro settings, and, to our knowledge, no studies have addressed this fundamental aspect in vivo. Moreover, several studies performed on samples from patients with ALL have failed to correlate ASNS expression levels with ASNase sensitivity (*9*, *10*), as reviewed in (*11*). To add to the controversy, knocking out *ASNS* did not sensitize melanoma cells to ASNase treatment in vivo (*12*), and ASNase-induced ASNS expression failed to prevent cell cycle arrest in ASNase-sensitive leukemic cells carrying Translocation-Ets-leukemia or ETV6/Acute Myeloid Leukemia 1 (*TEL*/*AML1*) fusion gene (*13*, *14*). Together, these observations call into question the clinical relevance of ASNS expression as a universal marker for predicting tumor sensitivity to ASNase treatment. Although many distinct cellular mechanisms involved in tumor cell adaptation to ASNase have been proposed over the past 2 decades (*15*–*22*), to which extent ASNS contributes to the resistance of ASNase-treated cancer cells in vivo remains unclear.

We recently established the proof of concept for the antitumor efficacy of ASNase using a preclinical model of non-Hodgkin’s B cell lymphoma (BCL), as well as in patients with diffuse large BCLs (DLBCLs) refractory to anti–CD20-based therapies (*21*). Here, we seek to uncover the impact of ASNS expression on the anticancer efficacy of ASNase in BCLs in vivo. We demonstrated that baseline ASNS expression in malignant B cells does not appear to be strongly associated with ASNase sensitivity. Using in vitro ^15^N(amide)-l-glutamine and in vitro and in vivo [U-^13^C_5_]-l-glutamine tracing, we showed that supraphysiological and physiological concentrations of asparagine prevent the detection of de novo asparagine synthesis in malignant cells, regardless of ASNS expression levels. Together, our results suggest that ASNS expression is required but not sufficient to predict the response of BCLs to ASNase and that the L-glutaminase activity of ASNase is indispensable for an optimal anticancer effect in B cell malignancies.

## RESULTS

### Supraphysiological concentration of asparagine prevents de novo asparagine biosynthesis in malignant B cells, regardless of ASNS expression level

**Fig. 1.**
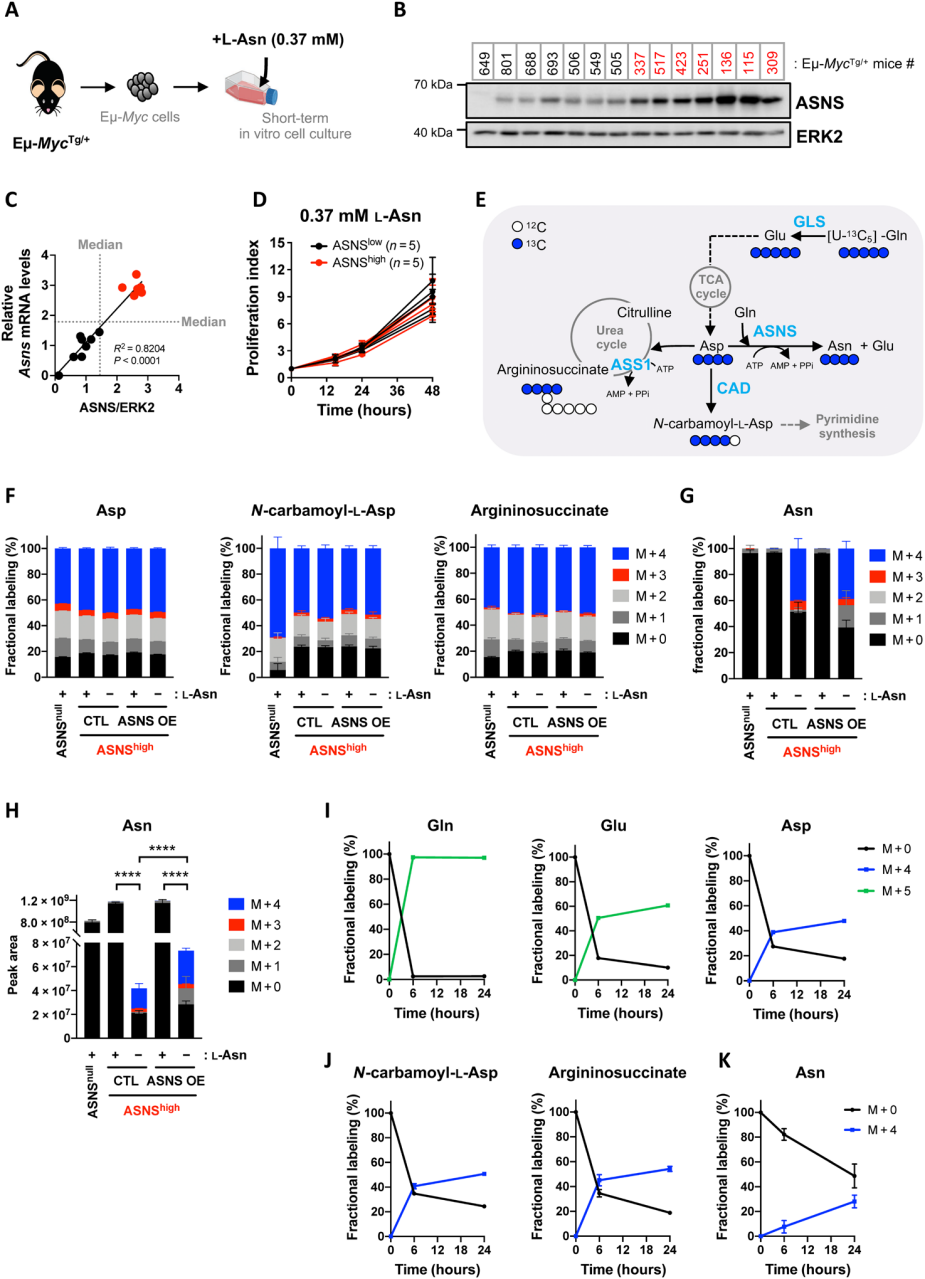
Supraphysiological concentration of asparagine prevents de novo asparagine biosynthesis, regardless of ASNS expression levels in primary malignant B cells. (**A**) Illustration of the experimental model. (**B**) Whole-cell lysates prepared from primary Eμ-*Myc* cells isolated from 14 Eμ-*Myc*^Tg/+^ mice were analyzed by immunoblot for ASNS and extracellular signal–regulated kinase 2 (ERK2) (loading control). (**C**) Correlation between relative levels of ASNS mRNA and protein in Eμ-*Myc* cells described in (B) (*n* = 3 independent experiments). The median of each variable (gray dotted lines) was used to classify Eμ-*Myc–*ASNS^null^ (*n* = 1), Eμ-*Myc*–ASNS^low^ (*n* = 7), and Eμ-*Myc*–ASNS^high^ (*n* = 6, red dots) cells. *R*^2^ and *P* value from simple linear regression test. (**D**) Proliferation index according to ASNS expression levels in Eμ-*Myc* cells (ASNS^low^, *n* = 5; ASNS^high^, *n* = 5) cultivated in the presence of l-asparagine (l-Asn) (0.37 mM) for 48 hours. Data expressed as means ± SD (*n* = 3 independent experiments). (**E**) Schematic representation of asparagine (Asn), *N*-carbamoyl-l-aspartate, and argininosuccinate biosynthesis from [U-^13^C_5_]-l-glutamine ([U-^13^C_5_]-Gln) through reactions catalyzed by ASNS, CAD (carbamoyl-phosphate synthetase 2, Asp transcarbamylase, and dihydroorotase), and argininosuccinate synthase 1 (ASS1), respectively. Aspartate (Asp) and Asp-derived metabolites produced by [U-^13^C_5_]-Gln oxidative metabolism have four labeled carbons (blue circles). ATP, adenosine 5′-triphosphate. (**F**) Fractional labeling of aspartate, *N*-carbamoyl-l-aspartate, argininosuccinate ^13^C isotopologues in Eμ-*Myc*–ASNS^null^ (#649) and Eμ-*Myc*–ASNS^high^ (#115) cells stably overexpressing V5-tagged murine ASNS (ASNS OE) or control vector (CTL), incubated with [U-^13^C_5_]-l-glutamine (2 mM) and with (+) or without (−) l-asparagine (0.37 mM) for 24 hours. Data expressed as means ± SD (*n* = 4 biological replicates). (**G** and **H**) Fractional labeling (G) and relative abundance (H) of asparagine ^13^C isotopologues in cells presented in (F). *P* value from *t* test; ****P < 0.0001. (**I** to **K**) Fractional labeling kinetics of unlabeled (M + 0) and fully carbon-labeled Gln, glutamate (Glu), and Asp (I), *N*-carbamoyl-l-aspartate and argininosuccinate (J), and Asn (K) in Eμ-*Myc*–ASNS^high^ (#115) cells incubated with [U-^13^C_5_]-l-glutamine (2 mM), in the absence of l-asparagine for 6 and 24 hours. Data are expressed as means ± SD (*n* = 3 biological replicates).

Using formalin-fixed paraffin-embedded biopsies at initial diagnosis DLBCL biopsies, we identified heterogeneous expression of ASNS in malignant B cells among patients (fig. S1A). In two large cohorts of patients with DLBCL (*23*, *24*), heterogeneous *Asns* mRNA expression was not associated with tumor burden or clinical factors associated with disease progression (table S1). To further understand the link between ASNS expression and de novo asparagine biosynthesis in tumor cells, we used the well-characterized Eμ-*Myc* mice model, in which *c-Myc* is expressed under the control of the μ-immunoglobulin heavy chain enhancer. We have previously demonstrated that spontaneous BCLs arising from the Eμ-*Myc^Tg/+^* mouse model share similar metabolic characteristics and metabolic heterogeneity with mature human BCLs, such as DLBCL (*21*). Primary malignant B cells (Eμ-*Myc* cells) harvested from BCLs of distinct Eμ-*Myc*^Tg/+^ transgenic mice were isolated and cultured for a short time in vitro (up to 3 weeks maximum). Therefore, to grow ASNS^null^– and ASNS^low^–Eμ-*Myc* cells in vitro and to assess their occurrence frequency in Eμ-*Myc*^Tg/+^ transgenic mice, primary Eμ-*Myc* cells were grown in Dulbecco’s modified Eagle’s medium (DMEM) supplemented with 0.37 mM l-asparagine, a concentration found in conventional and commercially available medium RPMI (Fig. 1A). RNA sequencing (RNA-seq) revealed an heterogeneous *Asns* mRNA expression among several independent Eμ-*Myc* cells harvested from 12 distinct Eμ-*Myc*^Tg/+^ mice, with Eμ-*Myc* #649 lacking *Asns* mRNA (fig. S1B). Heterogeneous levels of *Asns* transcript and ASNS protein were confirmed in 14 independent Eμ-*Myc* cells by real-time quantitative polymerase chain reaction (qPCR) with specific primers (fig. S1C) and by immunoblot (Fig. 1B), respectively. Moreover, *Asns* mRNA levels correlate with ASNS protein expression (Fig. 1C). On the basis of ASNS expression levels, we classified Eμ-*Myc* cells into ASNS^null^ (Eμ-*Myc* #649), ASNS^low^ (Eμ-*Myc* cells with ASNS protein or mRNA median expression around 1.0), and ASNS^high^ (Eμ-*Myc* cells with ASNS median expression around 2.5 to 3.0) (Fig. 1C). Consistent with asparagine auxotrophy, cell death was observed in Eμ-*Myc* cells lacking *Asns* mRNA expression (#649) incubated in the absence of extracellular l-asparagine, reinforcing the relevance of our in vitro culture system (fig. S1D). This result also suggests that extracellular sources of asparagine support the growth of ASNS^null^ BCLs in Eμ-*Myc*^Tg/+^ mice, thus challenging the importance of ASNS expression on the development of chemo-naïve BCLs.

ASNS has been reported to promote the growth of certain solid tumor cells such as colorectal (HCT116) and lung cancer (A549 and H460) cell lines (*25*, *26*). We therefore compared ASNS expression in four non-small cell lung (including A549) and five colon (including HCT116) carcinoma cell lines of human or murine origin with Eμ-*Myc* cells expressing low or high ASNS levels. Figure S1E shows that Eμ-*Myc*–ASNS^high^ cells (#136 and #115-CTL or overexpressing murine ASNS-V5) express ASNS levels comparable to solid cancer cell lines in which ASNS has been shown to be a rate-limiting enzyme for tumorigenesis (A549 and HCT116). Eμ-*Myc*–ASNS^low^ cells (clones 506 and 688) express lower levels of ASNS than all the other cell lines tested, reinforcing the relevance of our model to study ASNS function according to its expression level (fig. S1E). Last, consistent with the absence of nutritional stress in the in vitro setting used, Activating Transcription Factor 4 (ATF4) expression was not modulated among the clones, suggesting that the regulation of endogenous ASNS in Eμ-*Myc* cells is ATF4 independent (fig. S1F).

Consistent with clinical data from DLBCL patient samples (table S1), in vitro proliferation of Eμ-*Myc* cells with distinct ASNS expression was equivalent in asparagine-containing media, independent of ASNS expression levels (Fig. 1D and fig. S1, G and H), demonstrating the lack of association between ASNS expression levels and proliferation in BCLs. However, this might suggest either a weak contribution of de novo asparagine biosynthesis to cancer cell proliferation or a weak ASNS activity despite distinct expression levels. To test these hypotheses, we traced the incorporation of uniformly ^13^C-labeled glutamine ([U-^13^C_5_]-l-glutamine) into de novo asparagine synthesis catalyzed by ASNS, through liquid chromatography–mass spectrometry (LC-MS) analyses of cell extracts obtained from primary ASNS^null^– and ASNS^high^–Eμ-*Myc* cells stably expressing control vector (CTL) or V5-tagged murine ASNS (ASNS OE) (fig. S2A). Briefly, aspartate derived from oxidative metabolism of [U-^13^C_5_]-l-glutamine has four carbons labeled (M + 4). Aspartate is a key intermediate at the cross-roads of several anabolic reactions. Cytosolic enzymes such as ASNS, the multienzymatic protein CAD (carrying carbamoyl-phosphate synthetase 2, aspartate transcarbamylase, and dihydroorotase activities), and ASS1 (argininosuccinate synthase 1) use aspartate as a substrate to catalyze reactions synthetizing asparagine, the pyrimidine precursor *N*-carbamoyl-l-aspartate, or the urea cycle intermediate argininosuccinate, respectively (Fig. 1E). Thus, M + 4 carbon-labeled aspartate derived from the oxidative metabolism of [U-^13^C_5_]-l-glutamine generates asparagine, *N*-carbamoyl-l-aspartate, and argininosuccinate M + 4 isotopologues. In asparagine-containing medium, Eμ-*Myc* cells used oxidative metabolism of [U-^13^C_5_]-l-glutamine to synthetize carbon-labeled tricarboxylic acid (TCA) cycle metabolites, aspartate (M + 4), *N*-carbamoyl-l-aspartate (M + 4), and argininosuccinate (M + 4) (Fig. 1F), but they did not synthetize detectable levels of carbon-labeled asparagine, regardless of ASNS expression (Fig. 1G). In contrast, in asparagine-free medium, oxidative metabolism of [U-^13^C_5_]-l-glutamine contributed to de novo asparagine biosynthesis in Eμ-*Myc*–ASNS^high^ cells stably expressing control vector (CTL) and V5-tagged murine ASNS (ASNS OE), as shown by the detection of 40% asparagine M + 4 in average (Fig. 1G). The viability and proliferation of Eμ-*Myc*–ASNS^high^ cells (CTL or ASNS OE) were unaffected by the absence of asparagine in cell culture media for 24 hours (fig. S2B). These cells did not even induce ATF4 or ASNS protein expression despite the absence of extracellular asparagine, suggesting that nutritional vulnerabilities of ASNS^high^ Eμ-*Myc* cells are independent of environmental asparagine availability (fig. S2A). Thereby, for a similar level of ASNS protein (fig. S2A), our results demonstrate that extracellular asparagine dictates the dependence of Eμ-*Myc* cells on de novo asparagine biosynthesis. Unexpectedly, when analyzing total asparagine relative abundance, we observed a significant 28-fold decrease in intracellular asparagine in CTL–Eμ-*Myc*–ASNS^high^ cells displaying active de novo asparagine synthesis, as compared to their counterparts incubated in the presence of asparagine (Fig. 1H). Similarly, in the absence of asparagine, Eμ-*Myc*–ASNS^high^ cells overexpressing V5-tagged murine ASNS have 16-fold lower asparagine intracellular levels as compared to the same cells cultivated in medium supplemented with supraphysiological concentrations of asparagine. Nevertheless, overexpression of ASNS in these cells led to a significant increase in total intracellular asparagine levels compared to control cells (Fig. 1H). Using ^15^N(amide)-l-glutamine, which is directly incorporated into asparagine (M + 1) through the ASNS reaction, we confirmed that de novo asparagine biosynthesis is detected in Eμ-*Myc*–ASNS^high^ cells cultivated in the absence of asparagine, but not in Eμ-*Myc*–ASNS^high^ cells grown at supraphysiological asparagine levels (fig. S2, C and D). Oxidative metabolism of [U-^13^C_5_]-l-glutamine also contributed to de novo asparagine biosynthesis in Eμ-*Myc*–ASNS^high^ cells upon depletion of extracellular asparagine only, using ASNase (0.003 IU/ml) for 24 hours (Fig. 4B and fig. S2, E to G).

The passage of an enzymatic reaction product from one enzyme to the next is a very dynamic process when it occurs in the same cellular compartment. For instance, most reactions involved in the oxidative metabolism of glutamine are catalyzed by enzymes associated with each other at the mitochondrial inner membrane and mitochondrial matrix, allowing metabolic channeling of glutaminolysis products and TCA cycle intermediates. However, metabolic channeling can be interrupted along a metabolic pathway occurring in different cellular compartments. Glutamine-derived aspartate is synthetized through the reaction catalyzed by the aspartate aminotransferase Glutamic-Oxaloacetic Transaminase 2 (GOT2) in the mitochondria and then shuttles by active diffusion from the mitochondria to the cytoplasm, where it serves as a substrate for the synthesis of asparagine, arginine precursors, or pyrimidine precursors, through reactions catalyzed by ASNS, ASS1, and CAD, respectively. Although from glutamine to aspartate each enzyme is sequential in the pathway, metabolic channeling is interrupted when aspartate diffuses across the intermembrane space, a step that engages aspartate transporters. We therefore asked whether under active ASNS conditions, the flux of metabolic intermediates would be disturbed. In asparagine-free medium, tumor cell enrichment in [U-^13^C_5_]-l-glutamine and in glutamine-derived glutamate (M + 5) and aspartate (M + 4) peaked at 6 hours after addition of the tracer, consistent with a metabolic channeling of intermediates of oxidative metabolism of glutamine from glutaminase (GLS) to GOT2 (Fig. 1I). Unexpectedly, M + 4 isotopologues of *N*-carbamoyl-l-aspartate and argininosuccinate, which are synthetized in the cytoplasm from [U-^13^C_5_]-l-glutamine–derived aspartate M + 4, also peaked within 6 hours (Fig. 1J), but not asparagine M + 4, whose de novo synthesis kinetic is slower (Fig. 1K). This result suggested that the flux of aspartate toward ASNS is limited and is instead directed to other aspartate-consuming enzymes, a feature that is independent of the disruption of metabolic channeling. Moreover, in asparagine-free medium, the rates of cell enrichment in glutamate M + 5 and asparagine M + 4 differed, indicating that mitochondrial glutamine hydrolysis catalyzed by GLS might be the predominant metabolic route for glutamate synthesis and that ASNS, although active, might only weakly contribute to it (Fig. 1, I and K, and fig. S2H).

### Glutamine carbons minimally contribute to de novo asparagine biosynthesis in vivo in ASNS^high^-chemo-naïve BCLs and in mouse healthy pancreas

**Fig. 2.**
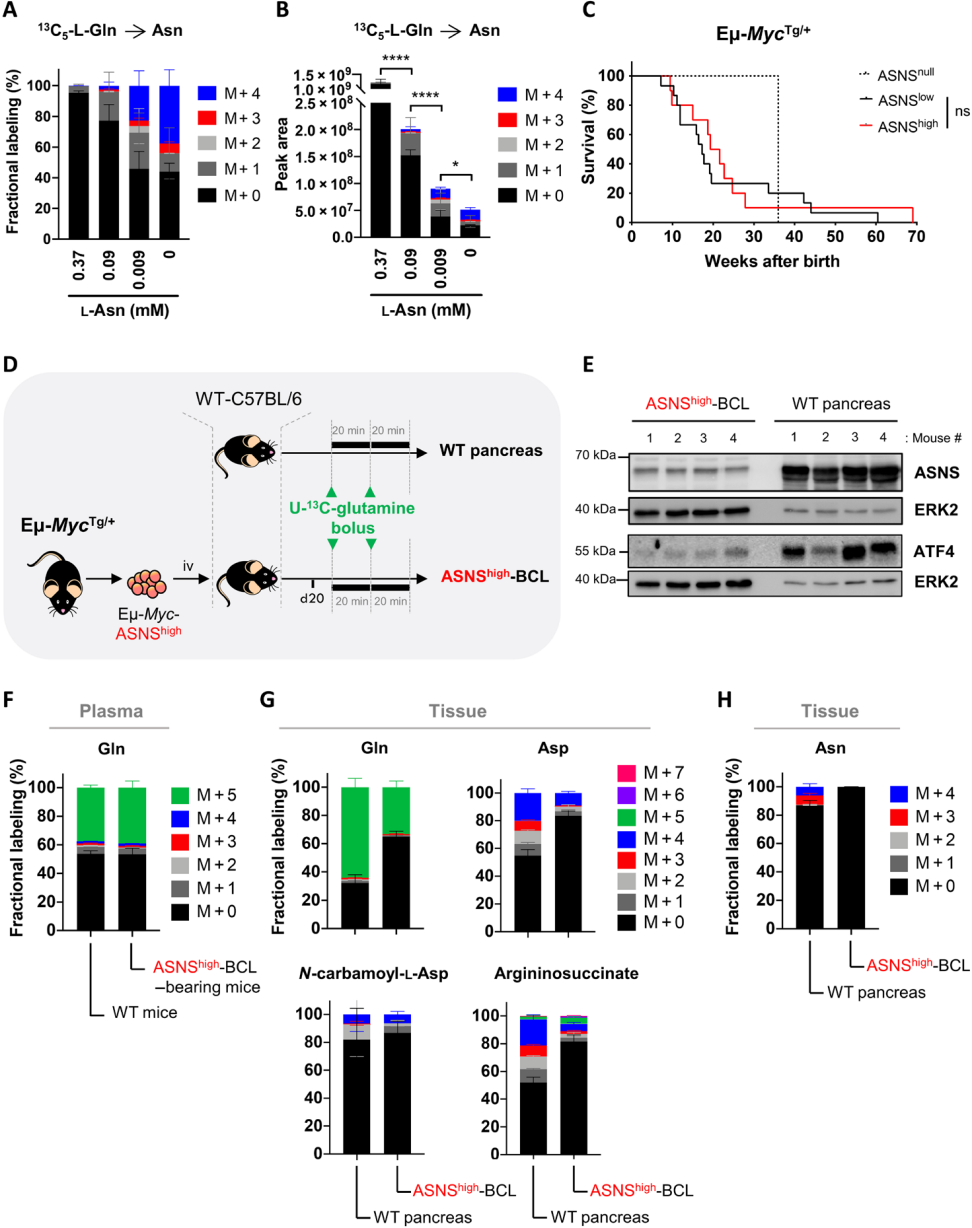
Environment dictates de novo asparagine biosynthesis in ASNS^high^ B cell lymphomas in vivo. (**A** and **B**) Fractional labeling (A) and peak area (B) of asparagine ^13^C isotopologues in primary Eμ-*Myc*–ASNS^high^ cells (from the Eμ-*Myc*^Tg/+^ mouse #115) incubated in the presence of [U-^13^C_5_]-l-glutamine (2 mM) and in the presence or absence of supraphysiological (0.37 mM), physiological (0.09 mM), and subphysiological (0.009 mM) l-asparagine concentrations for 24 hours. Data are expressed as means ± SD (*n* = 4 biological replicates). *P* value from *t* test; *P < 0.05; ****P < 0.0001. (**C**) Kaplan-Meier survival curves of Eμ-*Myc*^Tg/+^ transgenic mice according to ASNS protein expression levels in Eμ-*Myc* cells (ASNS^null^, *n* = 1; ASNS^low^, *n* = 15; ASNS^high^, *n* = 10). *P* value from log-rank test; ns, not significant. (**D**) Schematic representation of in vivo [U-^13^C_5_]-l-glutamine tracing. Primary Eμ-*Myc*–ASNS^high^ cells (from the Eμ-*Myc*^Tg/+^ mouse #115) were intravenously (iv) injected into WT C57Bl/6 mice. Upon BCL development, mice received two consecutive (of 20-min interval) discrete bolus of [U-^13^C_5_]-l-glutamine. Forty minutes following the first bolus, the plasma and ASNS^high^-BCLs were harvested, snap frozen, and analyzed by LC-MS for the detection of [U-^13^C_5_]-l-glutamine–derived metabolites. Following the same protocol, in vivo [U-^13^C_5_]-l-glutamine tracing was simultaneously performed in healthy WT C57Bl/6 mice to determine [U-^13^C_5_]-l-glutamine–derived metabolites in the pancreas. (**E**) Whole-cell lysates prepared from axillary ASNS^high^-BCLs and healthy pancreas were analyzed by immunoblot for the indicated proteins (*n* = 4 mice per group). ERK2 was used as a loading control. (**F**) Fractional labeling of glutamine ^13^C isotopologues in the plasma of WT C57Bl/6 mice (*n* = 5) and of ASNS^high^-BCL–bearing C57Bl/6 mice (*n* = 5), following two bolus of [U-^13^C_5_]-l-glutamine as described in (D). Data expressed as means ± SD. (**G** and **H**) Fractional labeling of glutamine (Gln), aspartate (Asp), *N*-carbamoyl-l-aspartate, argininosuccinate (G), and Asn (H) ^13^C isotopologues in healthy pancreas and in axillary ASNS^high^-BCLs, following two bolus of [U-^13^C_5_]-L-glutamine as described in (D). Data are expressed as means ± SD.

We next investigated whether de novo asparagine biosynthesis occurs under physiological and pathophysiological conditions. We first measured the concentration of asparagine in the plasma of wild-type (WT) immunocompetent C57Bl/6 mice and of C57Bl/6 mice bearing Eμ-*Myc*–ASNS^high^-BCLs (ASNS^high^-BCL). The average asparagine concentration in mouse plasma was 0.09 mM (with values ranging from 0.06 to 0.13 mM in WT animals) (fig. S3A), a value close to concentrations reported in human plasma samples (*27*). We next incubated Eμ-*Myc*–ASNS^high^ cells in DMEM medium supplemented with supraphysiological (0.37 mM), physiological (0.09 mM), or subphysiological concentrations (0.009 mM, i.e., 10-fold lower than the physiological concentration) of asparagine and in the presence of [U-^13^C_5_]-l-glutamine. When extracellular asparagine was added at physiological concentration, less than 3% of asparagine M + 4 was detected, indicating that glutamine carbons minimally contribute to de novo asparagine biosynthesis in malignant B cells, despite high ASNS expression (Fig. 2A). Nevertheless, in medium supplemented with subphysiological concentration of asparagine and in asparagine-free medium, cells synthetized asparagine from glutamine, with 23 and 38% of asparagine M + 4 detected, respectively (Fig. 2A), indicating that ASNS can be activated in these cells. Despite active de novo asparagine biosynthesis, total intracellular asparagine levels were lower (Fig. 2B). Our results indicate unappreciable levels of ASNS activity in Eμ-*Myc*–ASNS^high^ cells when mimicking the physiological concentration of extracellular asparagine in vitro, arguing for a lack of correlation between baseline ASNS expression and the levels of asparagine synthetized de novo by the cells. BCL development in chemo-naïve Eμ-*Myc*^Tg/+^ transgenic mice was similar regardless of ASNS expression levels (low or high) (Fig. 2C), in agreement with clinical data from patients with DLBCL (table S1). One hypothesis could be that circulating asparagine concentration is sufficient to sustain asparagine requirements of BCLs in vivo.

Tumor cell metabolism and proliferation are influenced by the nutritional composition of their local microenvironment. In several cancer mouse models, plasma concentrations of certain AAs vary from those found in the tumor interstitial fluid (TIF) (*28*). For that reason, we sought to determine in vivo the level of asparagine synthesis from carbon-labeled glutamine in mice bearing ASNS^high^-BCLs. As a positive control of endogenous ASNS activity, we also measured in vivo levels of asparagine synthesis in the organ with the highest ASNS expression (mRNA and protein) in mice and in humans, the pancreas (*29*). Therefore, Eμ-*Myc*–ASNS^high^ cells arising from Eμ-*Myc*^Tg/+^ mice (#115) were injected into the tail vein of syngeneic WT C57Bl/6 mice. Once BCL progression reached the ethical limit, ASNS^high^-BCL–bearing mice and healthy WT mice received two consecutive intraperitoneal bolus injections of [U-^13^C_5_]-l-glutamine at a 20-min interval. Forty minutes after the first injection of the tracer, plasma, ASNS^high^-BCLs, and the pancreas of tumor-free WT animals were collected, snap frozen, and then the metabolites from these samples were extracted and simultaneously analyzed by LC-MS (Fig. 2D). As expected, ASNS was highly expressed in healthy pancreas as compared to ASNS^high^-BCLs (Fig. 2E). This intense expression of ASNS is a consequence of elevated ATF4 expression in pancreatic cells (Fig. 2E) because of increased basal activation of protein kinase R (PKR)-like endoplasmic reticulum kinase (PERK) signaling, as others already described (*29*). In both healthy mice and ASNS^high^-BCL–bearing mice, [U-^13^C_5_]-l-glutamine enrichment achieved 40% in plasma (Fig. 2F). Substantial fully carbon-labeled glutamine (M + 5) was also detected in both tissue types (Fig. 2G). Although [U-^13^C_5_]-l-glutamine enrichment was lower in ASNS^high^-BCLs than in healthy pancreas (likely due to different rates of glutamine consumption and metabolism in these distinct tissues), 33% of [U-^13^C_5_]-l-glutamine was detected in ASNS^high^-BCLs (Fig. 2G), underscoring the in vivo glutamine addiction of lymphomas in the Eμ-*Myc* mouse model. Glutaminolysis, a two-step reaction catalyzed by GLS and GLUD (glutamate dehydrogenase), produces α-KG (α-ketoglutarate), which enters the TCA cycle as an M + 5 isotopologue (fig. S3B). From glutamine-derived α-KG (M + 5), oxidative metabolism of glutamine results in TCA cycle isotopologues with four-labeled carbons (M + 4), while reductive glutamine metabolism leads to a dominant citrate M + 5 isotopologue (fig. S3B). Aspartate is synthetized through transamination between oxaloacetate and glutamate, a reaction catalyzed by GOT2. While oxidative metabolism of glutamine from carbon-labeled glutamine leads to the synthesis of M + 4 isotopologues of aspartate and aspartate-derived metabolites (asparagine, *N*-carbamoyl-l-aspartate, and argininosuccinate), reductive glutamine metabolism results in M + 3 isotopologues (fig. S3B). In ASNS^high^-BCL, we observed glutamate M + 5, α-KG M + 5, and dominant M + 4 isotopologues of TCA cycle intermediates, indicating that oxidative metabolism of glutamine is the main metabolic route for TCA cycle anaplerosis in vivo (fig. S3C). Consistent with this, aspartate, *N*-carbamoyl-l-aspartate, and argininosuccinate M + 4 isotopologues were present at substantial levels (Fig. 2G), but carbon-labeled asparagine remained undetectable (Fig. 2H). This suggests that in ASNS^high^-BCLs in vivo, aspartate is directed to pyrimidine synthesis and the urea cycle but not toward de novo asparagine biosynthesis. Thus, asparagine synthesis might not be a predominant metabolic route from glutamine-derived aspartate in tumors, despite high ASNS expression. In the pancreas, both oxidative TCA cycle and reductive carboxylation of α-KG occur in vivo, as shown by the detection of M + 5 citrate and M + 3 aspartate from glutamine M + 5 (Fig. 2G and S3C), a feature we did not observe in ASNS^high^-BCLs. Moreover, in pancreas, aspartate M + 3 contributed to appreciable proportions of argininosuccinate M + 3 (8% without considering the proportion of isotopologues resulting from the urea cycling) (Fig. 2G) and of asparagine M + 3 (6.5%) (Fig. 2H). Through oxidative metabolism of glutamine, aspartate M + 4 also contributed to the synthesis of argininosuccinate M + 4 (19%) and to a lesser extent asparagine M + 4 (less than 6%) in vivo in the pancreas (Fig. 2, G and H). Overall, using two bolus injections of [U-^13^C_5_]-l-glutamine in mice, we were able to detect asparagine synthesis from oxidative and reductive metabolism of the tracer in the pancreas (12% of M + 3– and M + 4–labeled asparagine), further validating our in vivo stable isotope tracing approach. Nevertheless, given the incommensurable level of ASNS expressed in the pancreas, it could be argued that glutamine minimally contributes to de novo asparagine biosynthesis in pancreatic cells in vivo.

Together, our results demonstrate in vitro and in vivo that baseline ASNS expression levels do not reflect de novo asparagine biosynthesis. The microenvironmental concentration of asparagine may limit the flux of aspartate/glutamine through ASNS, severely questioning the relevance of ASNS expression as a marker to predict tumor response to treatment.

### ASNS expression reduces the survival of ASNase-treated BCL–bearing mice but is not sufficient to allow complete resistance to ASNase treatment in vivo

**Fig. 3.**
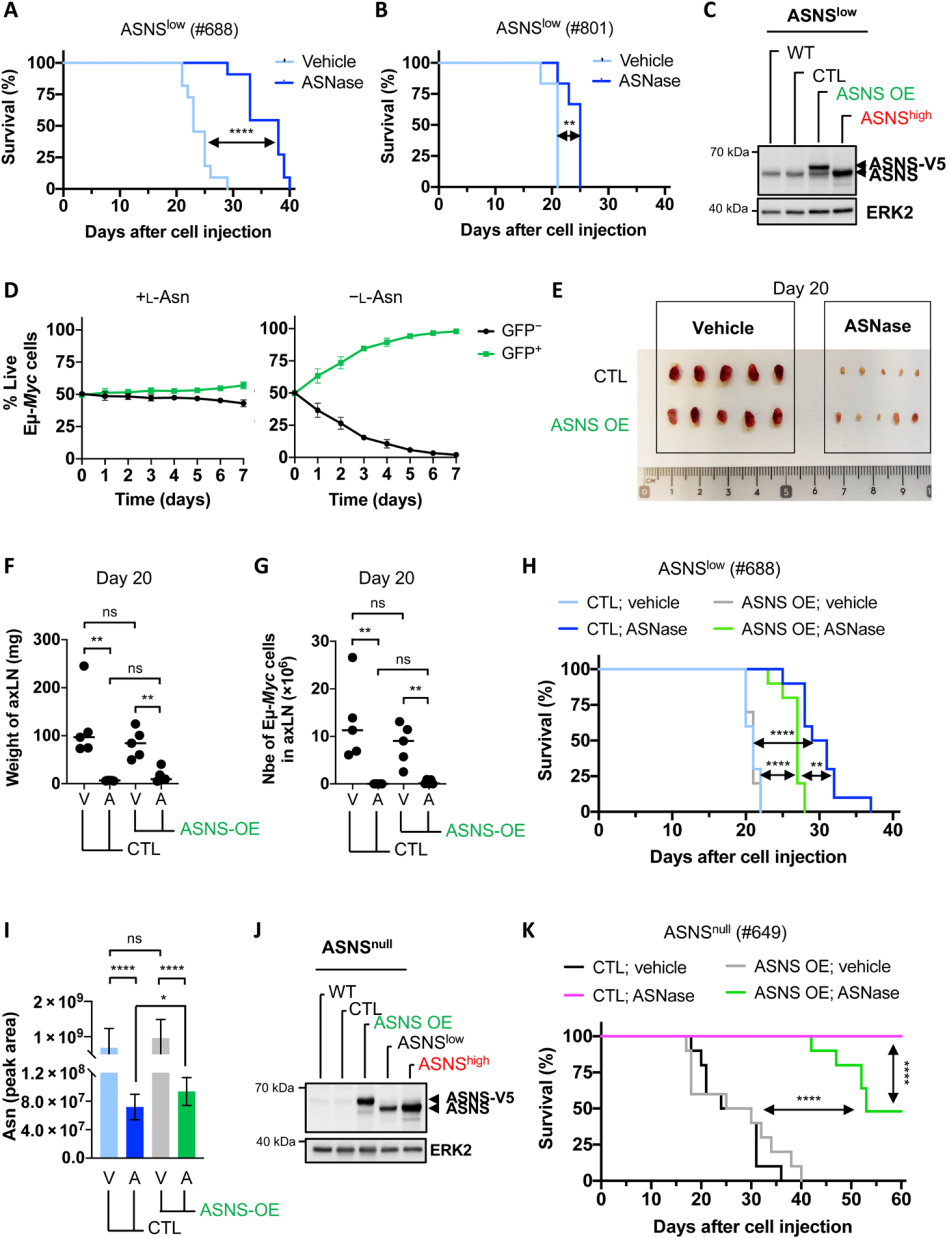
ASNS expression is not a robust marker of ASNase response in vivo in B cell lymphomas. (**A**) Survival curves of WT C57Bl/6 mice injected with Eμ-*Myc*–ASNS^low^ cells (from the Eμ-*Myc*^Tg/+^ mouse #688) and treated with vehicle or ASNase (2500 IU/kg) every 2 days, three times a week (*n* = 11 mice per group). *P* value from log-rank test. (**B**) As in (A) with Eμ-*Myc*–ASNS^low^ cells isolated from the Eμ-*Myc*^Tg/+^ mouse #801 (*n* = 6 mice per group). (**C**) Whole-cell lysates prepared from Eμ-*Myc*–ASNS^low^ cells (#688) WT or overexpressing control vector (CTL) or V5-tagged murine ASNS (ASNS OE), and Eμ-*Myc*–ASNS^high^ cells (#115) were analyzed by immunoblot for the indicated proteins. ERK2, loading control. (**D**) Competition assay of live Eμ-*Myc*–ASNS^low^ cells stably overexpressing [green fluorescent protein–positive (GFP^+^)] or not (GFP^−^) V5-tagged murine ASNS, in media supplemented with (+) or not (−) l-Asn (0.37 mM). Data are expressed as means ± SD (*n* = 3 independent experiments). (**E**) Axillary lymph node (axLN) phenotype (*n* = 5 mice per group) 20 days following injection of Eμ-*Myc*–ASNS^low^ cells presented in (C). From days 7 to 20, mice received vehicle or ASNase (2500 IU/kg) as described in (A) (*n* = 5 mice per group). (**F** and **G**) Weight of axLN (F) and number of live Eμ-*Myc* cells (GFP^+^) in axLN (G) presented in (E) (*n* = 5 mice per group). *P* value from *t* test. (**H**) Survival curves of WT C57Bl/6 mice injected with Eμ-*Myc*–ASNS^low^ cells presented in (C) and treated with vehicle or ASNase (2500 IU/kg) as in (A) (*n* = 10 mice per group). *P* value from log-rank test. (**I**) Relative abundance of intracellular asparagine in axillary BCLs harvested at end point from mice presented in (H) (*n* = 7 mice per group). *P* value was from *t* test. (**J**) As in (C) with Eμ-*Myc*–ASNS^null^ cells (#649). (**K**) As in (H) with Eμ-*Myc*–ASNS^null^ cells presented in (J). ASNase (2500 IU/kg) was administered for 7.5 weeks as described in (A) (*n* = 10 mice per group). *P* value from log-rank test. *P < 0.05; **P < 0.01; ***P < 0.001; ****P < 0.0001.

ASNS has long been considered as a marker of cancer cell’s sensitivity to ASNase in hematological malignancies and more recently in solid cancers. Faced with highly controversial results in the field, we decided to analyze the effect of ASNase treatment in the development of Eμ-*Myc*–BCL expressing low levels of ASNS in vivo. We selected Eμ-*Myc*–ASNS^low^ cells isolated from four independent Eμ-*Myc*^Tg/+^ transgenic mice (#504, #506, #688, and #801) based on their low and comparable ASNS protein expression levels in axillary BCLs in vivo (fig. S4A). Notably, ASNase treatment led to different anticancer responses (Fig. 3, A and B, and fig. S4, B and C). To further validate our observations in the same genetic background, we measured the impact of ASNS overexpression in Eμ-*Myc*–ASNS^low^ cells. Primary Eμ-*Myc*–ASNS^low^ lymphoma cells (harvested from Eμ-*Myc*^Tg/+^ mice #688) were transduced with retroviral green fluorescent protein (GFP)–encoded vector (CTL) or GFP-encoded m*Asns-v5* vector to overexpress V5-tagged murine ASNS (ASNS OE). ASNS expression levels in Eμ-*Myc*–ASNS^low^–ASNS OE cells were equivalent to those observed in Eμ-*Myc*–ASNS^high^ cells (Fig. 3C and fig. S4D). When cultivated in medium supplemented with supraphysiological concentration of asparagine, the proliferation rate of Eμ-*Myc*–ASNS^low^–CTL cells was equivalent to that of Eμ-*Myc*–ASNS^low^–ASNS OE cells (Fig. 3D, left). However, in asparagine-free medium, Eμ-*Myc*–ASNS^low^–ASNS OE cells showed a competitive growth advantage in vitro over Eμ-*Myc*–ASNS^low^–CTL cells (Fig. 3D, right), indicating that the overexpressed murine ASNS-V5 form is active and fully functional upon asparagine depletion. We next asked whether ASNS was able to protect tumor cells from plasma asparagine depletion in vivo. To address this question, we intravenously injected stable Eμ-*Myc*–ASNS^low^ cells expressing either control vector (CTL) or vector encoding V5-tagged murine ASNS (ASNS OE) into syngeneic C57Bl/6 mice. Seven days later, mice received vehicle (NaCl 0.9%) or ASNase treatment (2500 IU/kg) every other day, three times a week. Twenty days after tumor cell transfer, mice from all groups were euthanized, and enlarged lymph nodes and spleen were harvested, weighed, and analyzed by flow cytometry to determine the percentage of malignant Eμ-*Myc* cells (GFP^+^) in these secondary lymphoid organs. The dose and frequency of ASNase administration did not affect animals’ weight (fig. S4E). In vehicle-treated mice, overexpression of murine ASNS-V5 did not have an impact on the development of BCLs (Fig. 3, E and F, and fig. S4, F to H). However, ASNase treatment considerably reduced BCL development, regardless of ASNS expression. This was shown by the smaller size and weight of lymph nodes and spleen in all ASNase-treated mice than in vehicle-treated mice (Fig. 3, E and F, and fig. S4, F to H). In agreement, the number of live Eμ-*Myc* cells (GFP^+^) in axillary lymph nodes and in the spleen was significantly lower in ASNase-treated mice, regardless of ASNS expression (Fig. 3G and fig. S4I).

We then repeated the same experiment to compare the survival of mice until BCL development reached the ethical limit. Overexpression of murine ASNS-V5 did not affect the development of ASNS^low^-BCL in vehicle-treated mice (Fig. 3H). However, as expected, it only partially reduced the survival of ASNase-treated mice, despite further induction of endogenous ASNS expression (Fig. 3H and fig. S4J). This partial protection correlates with a significantly higher total asparagine level under ASNase treatment in ASNS^low^-BCLs overexpressing ASNS compared to that in control ASNS^low^-BCLs (Fig. 3I). Given that ASNS expression is responsive to ASNase, we took advantage of the Eμ-*Myc*–ASNS^null^ cells (#649), which cannot adapt with respect to ASNS expression in response to ASNase. Overexpression of murine ASNS-V5 (ASNS OE) in these cells was equivalent to endogenous levels of ASNS expressed in Eμ-*Myc*–ASNS^high^ cells (Fig. 3J). We therefore injected CTL– and ASNS OE–Eμ-*Myc*–ASNS^null^ cells into syngeneic C57Bl/6 mice and treated them with vehicle or ASNase (2500 IU/kg) every 2 days, three times a week for 7.5 weeks. Consistent with our findings (Fig. 3, E to H), overexpression did not influence the development of ASNS^null^-BCL (Fig. 3K). While 100% of mice bearing Eμ-*Myc*–ASNS^null^ (CTL) cells showed a complete response to ASNase, the survival of ASNase-treated mice bearing Eμ-*Myc*–ASNS^null^–ASNS OE was significantly increased, and 50% of those mice did not develop BCLs during the therapy (Fig. 3K). Together, our results demonstrate that ASNS expression can only partially protect BCLs from ASNase treatment in vivo.

### Asparaginase activity alone is not sufficient to ensure an optimal outcome of ASNase therapy in ASNase-sensitive BCLs

**Fig. 4.**
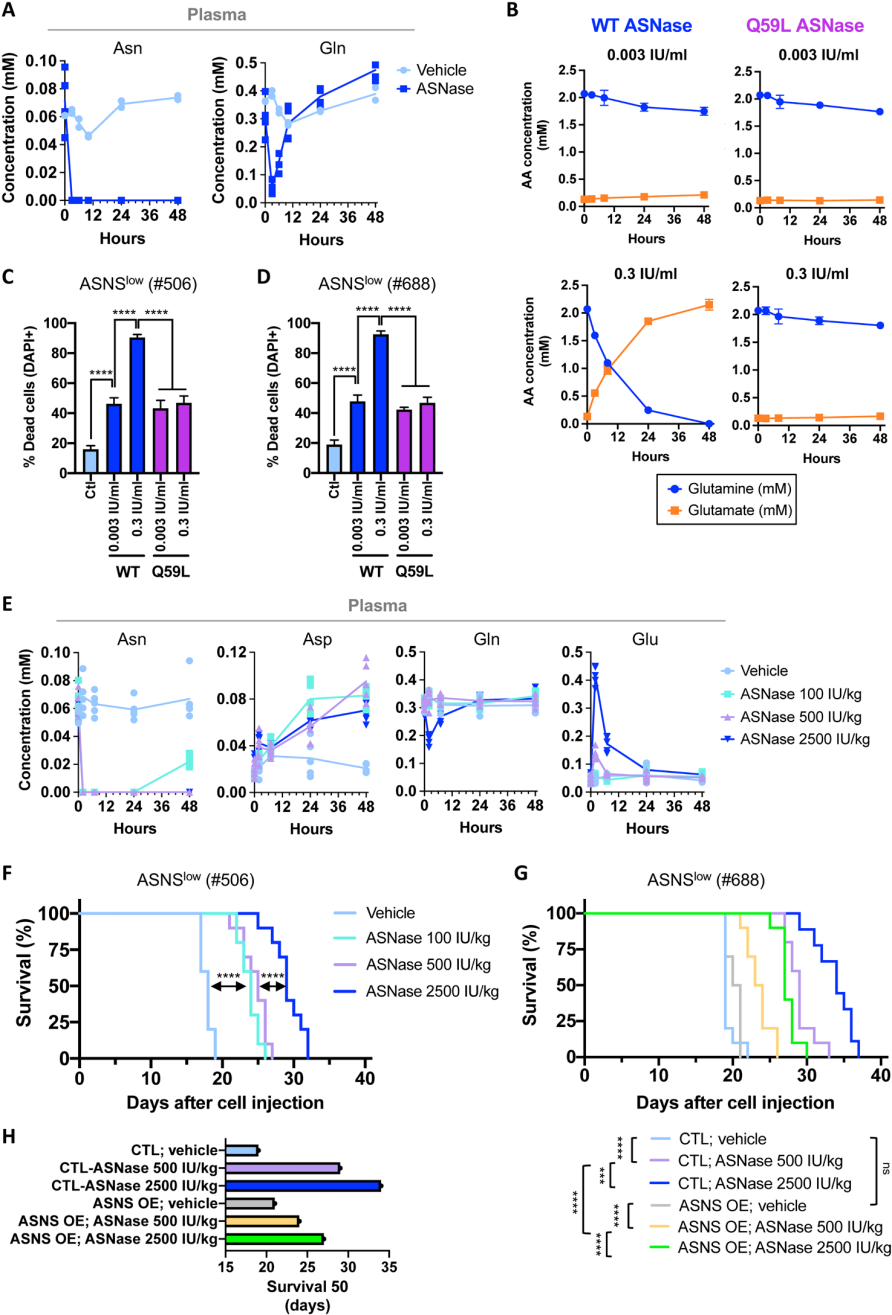
ASNase’s glutaminase activity is associated with an optimal in vivo outcome in ASNase-sensitive B cell lymphoma–bearing mice. (**A**) Plasma concentrations of asparagine (Asn) and glutamine (Gln) over time (0, 3, 6, 10, 24, and 48 hours) following a single intraperitoneal injection of vehicle (NaCl 0.9%; *n* = 2) or ASNase (2500 IU/kg; *n* = 4) in WT C57Bl/6 mice. For time 0, blood was immediately collected before ASNase injection. (**B**) Effect of recombinant WT or mutant Q59L ASNase over time on glutamine and glutamate concentrations in DMEM supplemented with l-glutamine (2 mM) and l-asparagine (0.37 mM). (**C** and **D**) Percentage of dead Eμ-*Myc*–ASNS^low^ cells [from the Eμ-*Myc*^Tg/+^ #506 mouse (C) and Eμ-*Myc*^Tg/+^ #688 mouse (D)] incubated in DMEM supplemented with l-glutamine (2 mM) and l-asparagine (0.37 mM), with or without (CTL) WT ASNase or mutant ASNase Q59L for 48 hours. Data are expressed as means ± SD (*n* = 3 independent experiments). *P* value from *t* test; ****P < 0.0001. (**E**) Plasma concentrations of asparagine (Asn), aspartate (Asp), glutamine (Gln), and glutamate (Glu) over time (0, 2, 7, 24, and 48 hours) following a single injection of ASNase (100, 500, or 2500 IU/kg) in Eμ-*Myc*–ASNS^low^–bearing mice (*n* = 5 mice per group). (**F**) Survival curve of WT C57Bl/6 mice intravenously injected with primary Eμ-*Myc*–ASNS^low^ cells (from the Eμ-*Myc*^Tg/+^ mouse #506) and treated with vehicle or ASNase 100 IU/kg daily or 500 or 2500 IU/kg every 2 days, three times a week (*n* = 10 mice per group). *P* value from log-rank test; ****P < 0.0001. (**G**) As in (F), with Eμ-*Myc*–ASNS^low^ cells (from the Eμ-*Myc*^Tg/+^ mouse #688) stably overexpressing control vector (CTL) or V5-tagged murine ASNS (ASNS OE). ASNase (500 or 2500 IU/kg) was administered every 2 days, three times a week (*n* = 10 mice per group; CTL-ASNase 2500 IU/kg, *n* = 9 mice). *P* value from log-rank test; ***P < 0.001; ****P < 0.0001. (**H**) Days (from tumor cell injection) corresponding to mice survival 50% presented in (G).

The lack of consistent results regarding ASNS expression and the anticancer effect of ASNase may be due to the l-glutaminase activity of clinically approved *Escherichia coli* type II ASNases, although their affinity for glutamine is much lower than that for asparagine. We sought to investigate in vivo the anticancer effect of ASNase with ASNase activity alone or combined with its l-glutaminase activity. When administered once by intraperitoneal injection into immunocompetent C57Bl/6 mice, ASNase (2500 IU/kg) induced a rapid and sustained depletion of plasma asparagine levels that remained below the limit of detection from 3 hours and up to 48 hours after injection (Fig. 4A). Circulating glutamine concentration in plasma of the same animals rapidly decreased by 80% 3 hours after ASNase injection. In contrast to asparagine, this reduction was only transient, and glutamine concentration returned to baseline levels 12 hours after injection (Fig. 4A). To uncover the respective contribution of ASNase activities (ASNase activity alone or ASNase/l-glutaminase combined) on cell death, we treated Eμ-*Myc*–ASNS^low^ cells with recombinant *E. coli* ASNase WT (0.003 or 0.3 IU/ml) or mutant Q59L lacking the l-glutaminase activity (*30*, *31*). As presented in Fig. 4B, the low dose of WT and Q59L ASNase (0.003 IU/ml) failed to convert glutamine into glutamate. When used at a higher dose (0.3 IU/ml), WT ASNase catalyzes glutamine conversion into glutamate, but the ASNase Q59L mutant does not. Hydrolysis of extracellular asparagine and glutamine with WT ASNase (0.3 U/ml) promoted massive cell death (>90%), whereas depletion of asparagine only (using the ASNase Q59L mutant or the low dose of WT ASNase) resulted in significantly less cell death (Fig. 4, C and D). The cytotoxic effect of the low dose of WT ASNase (0.003 IU/ml) could be prevented by addition of high extracellular asparagine concentrations, presumably by overcoming ASNase activity (fig. S5A). Short-time treatment (4 hours) of Eμ-*Myc*–ASNS^low^ cells with high dose of ASNase (1 IU/ml), which decreased extracellular l-glutamine concentration by 70% without significant cellular toxicity (fig. S5, B and C), reduced the abundance of TCA cycle metabolites and of most precursors of nucleotide synthesis (fig. S5D), suggesting that the dual activity of ASNase rapidly affects mitochondrial metabolism and nucleotide synthesis.

To investigate this further in vivo, we administered different amounts of ASNase (100, 500, and 2500 IU/kg). At 500 and 2500 IU/kg, circulating asparagine was stably depleted after 2 hours and up to 48 hours after ASNase injection. At 100 IU/kg, circulating asparagine was depleted as efficiently as with higher doses during the first 24 hours, but then its level gradually increased (Fig. 4E). Consistent with hydrolysis of asparagine in the circulation, plasma aspartate concentration increased, with no significant difference between ASNase doses (Fig. 4E). After injection of ASNase (100 and 500 IU/kg), plasma glutamine concentration did not decrease (Fig. 4E). As expected, 2 hours after injection of 2500 IU/kg, a rapid and short-live decrease in plasmatic glutamine concentration is accompanied by an accumulation of glutamate, which returns to basal levels within 24 hours (Fig. 4E). While no significant change in glutamine concentration was observed after injection of 500 IU/kg, glutamate concentration increased slightly but significantly, suggesting that concentration of circulating glutamate might be a highly sensitive indicator of ASNase’s l-glutaminase activity in vivo. Accordingly, the survival of ASNase-sensitive ASNS^low^-BCL–bearing mice treated with ASNase (100 and 500 IU/kg) was significantly lower than that of mice treated with ASNase (2500 IU/kg) (Fig. 4F). To further support our conclusions, mice bearing Eμ-*Myc*–ASNS^low^ cells (CTL and ASNS OE) used in Fig. 3H were treated with ASNase (500 or 2500 IU/kg) as previously described. This experiment indicated that the depletion of plasma asparagine only was less effective in prolonging mouse survival than the depletion of both asparagine and glutamine, independently of ASNS expression (Fig. 4G and H). This result suggests that the l-glutaminase activity of ASNase prevents resistance, at least in part because glutamine is a direct substrate of ASNS (fig. S2, C and D). Together, our data indicate that the asparaginase activity alone is less efficient than the combination of asparaginase and l-glutaminase activities, which provides optimal antitumor effects in ASNase-sensitive BCLs-bearing mice.

## DISCUSSION

With the multiplication of multiomic studies, authors frequently conclude that a given metabolic pathway is modulated solely on the basis of the expression of one, or a group, of metabolic enzymes in a same cell population. Unfortunately, this is not always true. For instance, here we would have incorrectly concluded that a subset of malignant B cells expresses high ASNS levels to synthesize asparagine, while their counterparts expressing low ASNS do not. Similarly, total levels of intracellular metabolites do not necessarily reflect the activity of their biosynthetic enzyme but may also be a consequence of cellular uptake or accumulation due to reduced activity of downstream metabolic pathways (*32*). Our study highlighted the importance of assessing metabolism using appropriate optimized methods such as in vitro and in vivo tracing of nutrients labeled with a stable isotope, to provide high-quality data and reliable interpretations of downstream metabolic pathways. By performing in vivo [U-^13^C_5_]-l-glutamine tracing, we revealed that glutamine carbons minimally contribute to de novo asparagine biosynthesis in ASNS^high^-BCLs and in healthy pancreatic cells, which express incommensurable levels of ASNS (Fig. 2). Thus, the ability of mammalian cells to produce asparagine in vivo is negligible despite high levels of ASNS, indicating a lack of correlation between ASNS expression and activity under physiological and pathological conditions.

The physiological concentration of asparagine in human and murine plasma (<0.1 mM) is one of the lowest among all AAs and seems to be equivalent between plasma and TIF in a mouse model of pancreatic adenocarcinoma (*28*). Our study demonstrates that supraphysiological and physiological concentrations of extracellular asparagine prevent its de novo cellular biosynthesis via ASNS (Figs. 1, F to H, and 2). The composition of commercially available media greatly differs in terms of asparagine concentration. For instance, cancer cell lines grown in RPMI medium (which contains a supraphysiological concentration of asparagine) are likely to be unable to synthetize asparagine de novo, regardless of ASNS expression, whereas cell lines grown in DMEM (which lacks asparagine) will synthetize asparagine de novo according to endogenous ASNS expression levels. In line with previous publications (*33*, *34*), our study further supports that it is of utmost importance to pay attention to the composition of the medium when studying AA metabolism in cancer, especially if comparing independent cancer cell lines from the same tumor entity that are to be grown in different cell culture media.

We observed that Eμ-*Myc*–ASNS^high^ cells cultivated in asparagine-free medium still exhibit asparagine shortage despite detectable levels of glutamine-derived aspartate and active de novo asparagine biosynthesis (Figs. 1F and 2B). This unexpected observation was also made in a mouse model of breast cancer metastasis (*19*). In vivo, lower levels of total intracellular asparagine detected in ASNase-treated ASNS^low^-BCLs seem to be sufficient to meet the requirements of malignant B cell proliferation.

Lack of ASNS expression due to hypermethylation of its promoter is a well-known hallmark of ALL (*20*, *35*). Here, in chemo-naïve BCLs arising from the Eμ-*Myc* mouse model and in biopsies from newly diagnosed DLBCL, we identified heterogeneous ASNS expression (predominantly ASNS^low^ or ASNS^high^) (Fig. 1, A to C, and fig. S1). In contrast to ALL, the frequency of ASNS^null^-BCL development is rare (fig. S1). Baseline ASNS expression in Eμ-*Myc*–BCLs is transcriptionally regulated, but independently of ATF4. Although our study did not aim to investigate the mechanisms by which BCLs express low or high levels of ASNS, epigenetic modifications in the ASNS promoter cannot be ruled out. However, why, in the absence of acute nutritional stress (such as the one induced by ASNase), would malignant cells express different levels of ASNS? Our study indicates that de novo asparagine biosynthesis does not seem to be the predominant role of ASNS in malignant tissues in vivo. Many metabolic enzymes have “moonlight” functions (*36*–*40*). In several human proliferating cancer cell lines and in yeast, ASNS was found to be recruited, around the centrosome, to the mitotic spindle (*41*, *42*). Upon ASNase treatment, this colocalization is lost as cells might require ASNS to regain its enzymatic function, which suggests that ASNS might have a moonlight role in cell division (*41*).

With the growing interest in ASNase therapy in several hematological malignancies and solid cancers, knowing if ASNS expression can predict tumor response to ASNase is of great clinical interest. Many observations suggest that ASNS may not be a universal marker of ASNase susceptibility (*9*, *10*, *12*), as reviewed in (*11*). We therefore investigated the anticancer effect of ASNase in vivo in BCLs expressing equivalent low ASNS levels. Only two out of the four ASNS^low^-BCLs were sensitive to the treatment. Many factors other than ASNS have been described as influencing ASNase susceptibility (*15*, *17*, *19*, *21*, *43*–*45*). While the exact mechanism(s) of resistance in each ASNase low-sensitive ASNS^low^-BCL remains to be determined, this is beyond the scope of the present study. However, we could establish that ASNS overexpression in ASNS^low^–Eμ-*Myc* cells sensitive to ASNase treatment did not accelerate *Myc*-driven BCL growth (Fig. 3). Similarly, ASNS does not affect primary tumor growth but rather drives invasion and metastasis formation in a mouse model of breast cancer (*4*). Our results and those of others (*4*, *46*) are not consistent with those obtained in a genetically engineered mouse model of mutant KRAS-driven NSCLC, in which ASNS has been shown to be rate limiting for tumorigenesis (*26*). This might be explained by the influence of KRAS on AA concentrations in the TIF, making chemo-naïve lung cancer cells dependent on de novo asparagine synthesis to sustain proliferation.

Several studies demonstrated that the l-glutaminase activity of ASNase is a determinant of its cytotoxic effects on leukemic cells in vitro (*47*), and of its antitumor effects in vivo, especially in cancer cell expressing endogenous ASNS levels (*30*, *31*, *48*). By administrating ASNase at different doses and frequencies, we massively reduced its l-glutaminase activity while preserving the long-lived depletion of circulating asparagine and observed that ASNase-sensitive BCLs develop significantly faster upon this treatment (Fig. 4, E to H). Although at 2500 IU/kg ASNase induces transient depletion of circulating glutamine, this is associated with a significant improvement of the overall survival of ASNase-sensitive BCL-bearing mice (Fig. 4, E to H). Unfortunately, there is no consensus methodology for the use of ASNase (type and posology) in vivo, which makes it difficult to compare studies, especially those aimed at determining the contribution of ASNS to ASNase sensitivity. The questions of how much asparaginase and what source of asparaginase are needed to achieve optimal results with low toxic side effects remain open. Different types of ASNase differ in their affinity for glutamine, with Erwinaze (*Erwinia chrysanthemi*) having the highest l-glutaminase activity compared to native *E. coli* ASNase (*47*). Therefore, it would be crucial to determine the l-glutaminase activity of new ASNases currently on the market (Spectrila and Oncaspar). For this reason, our study also highlighted the importance of measuring circulating glutamine and glutamate concentration as an indicator of ASNase l-glutaminase activity in mice, a finding that could also be considered in the clinics.

## MATERIALS AND METHODS

### Mice

C57BL/6 Eμ-*Myc* transgenic mice (Eμ-*Myc*^Tg/+^) were purchased from the Jackson Laboratory (002728) and are bred and maintained at our local animal facility (C3M, INSERM U1065, Nice, France). C57BL/6JOlaHsd females were purchased from Envigo and housed at our local animal facility (C3M, INSERM U 1065, Nice, France, B 06-088-20). All mice were maintained in specific pathogen–free conditions, and all experimental procedures were performed in compliance with the protocols approved (no. 05111-02 and no. APAFIS#25228-2020032917261458 v9) by the Institutional Animal Care and Use Committee (University Nice, Côte d’Azur) and the Ministère de la Recherche et de l’Innovation.

### Cell lines and culture conditions

Primary malignant B cells overexpressing *c-Myc* (Eμ-*Myc* cells) were isolated from BCLs of different Eμ-*Myc*^Tg/+^ transgenic mice as previously described (*37*) and cultivated for a short time in vitro in DMEM-GlutaMAX media (31966047, Thermo Fisher Scientific) supplemented with 10% fetal bovine serum (FBS; F7524, Sigma-Aldrich), 50 μM 2-mercaptoethanol (31350010, Thermo Fisher Scientific), 0.37 mM l-asparagine (A0884, Sigma-Aldrich), 10 mM Hepes pH 7.4 (15630056, Thermo Fisher Scientific), and penicillin-streptomycin (100 U/ml) (15140122, Thermo Fisher Scientific).

293T cells [CRL-1573, American Type Culture Collection (ATCC)] were maintained in DMEM-GlutaMAX media supplemented with 10% FBS and penicillin-streptomycin (100 U/ml) and used to produce VSV-G retroviral vectors.

The human non-small cell lung cancer cell lines A549 (CCL-185, ATCC) and H1975 (CRL-5908, ATCC) and the murine Lewis lung carcinoma cell line (CRL1642, ATCC) were maintained in DMEM-GlutaMAX media (31966047, Thermo Fisher Scientific) supplemented with 10% FBS and penicillin-streptomycin (100 U/ml). The murine KRAS^G12D^-p53^−/−^ (KP) lung cancer cells established in our laboratory by S.M. from the KRAS^G12D^-p53^−/−^ mice bred and maintained at our animal facility were cultivated in RPMI-GlutaMAX media (61870044, Thermo Fisher Scientific) supplemented with 10% FBS and penicillin-streptomycin (100 U/ml).

The human colon carcinoma cell lines LS174T (CL-188, ATCC), T84 (CCL248, ATCC), HT29 (HTB-38, ATCC), and HCT116 (CCL-247) were maintained in DMEM-GlutaMAX media (31966047, Thermo Fisher Scientific) supplemented with 10% FBS and penicillin-streptomycin (100 U/ml). The murine colon carcinoma cell line CT26 (CRL-2638, ATCC) was maintained in RPMI-GlutaMAX (61870044, Thermo Fisher Scientific) supplemented with 10% FBS and penicillin-streptomycin (100 U/ml). All cell lines are mycoplasma free and routinely tested for mycoplasma.

To assess baseline ASNS expression in solid cancer cell lines, malignant cells cultivated in DMEM media were incubated for 4 days in the presence of l-asparagine (0.37 mM). Next, each cell line was seeded at 1.10^6^ cells in 100-mm dishes and incubated for 24 hours in its respective media, RPMI or DMEM media, the latter being supplemented with l-asparagine (0.37 mM).

### DLBCL patient samples

Newly diagnosed DLBCL samples from patients were selected from cohorts previously described (*21*). The patients received the necessary information, and consent was obtained as previously described (*21*). The morphologic classification of the tumors was carried out according to the World Health Organization criteria (*49*).

### Data analysis

We used the R-CHOP cohort from the GSE10846 study (*23*) as previously described (*21*). RNA-seq alignment files and clinical information from the EGAS00001002606 study (*24*) were downloaded from the European Genome-Phenome Archive. Quantification of genes was performed using featureCounts v1.5.0-p3 based on Ensembl GTF release 75 annotations. Samples with less than 1 M of sequenced reads were removed. RNA-seq data were then normalized using the Bioconductor package DESeq2.

For each cohort, patient samples were divided into *Asns* high and low groups (above and below the median) on the basis of their respective *Asns* expression levels. Statistical significance was assessed by Student’s *t* test or chi-square test.

### ASNase enzyme and variants

For in vitro and in vivo studies, we used the native *E. coli* ASNase II from Jazz Pharmaceutical (Kidrolase). For Fig. 4 (B to D), we used *E. coli* ASNase II recombinant proteins, WT, and mutant Q59L ASNase produced as previously described (*30*).

### In vivo transfer of Eμ*-Myc* cells and survival experiments

The transfer of mouse primary Eμ-*Myc* lymphoma cells was performed into syngeneic, nontransgenic, 6-week-old C57BL/6JOlaHsd females (Envigo) by intravenous injection of 100,000 live cells per recipient mouse. Seven days after cell injection, mice received native *E. coli* ASNase (Kidrolase, Jazz Pharmaceutical) as follows: 100 IU/kg every day or 500 IU/kg or 2500 IU/kg every 2 days, three times a week, by intraperitoneal injection. Vehicle-treated mice were intraperitoneally injected with NaCl 0.9% every 2 days, three times a week. Tumor onset was determined by inguinal lymph node palpation. Food was given ad libitum. No randomization was performed. ASNase-treated mice were euthanized 2 to 4 hours following a last ASNase injection. Eμ-*Myc*–bearing animals were euthanized by cervical dislocation. Blood was then collected through cardiac punction, placed in lithium heparinate–coated tubes (BD Microtainer), and centrifuged 6000*g* for 2 min to isolate the plasma. The plasma samples were collected, snap frozen, and stored at −80°C before being analyzed by LC-MS to quantify plasma concentrations of AAs. To study the survival of Eμ-*Myc*–bearing mice following Eμ-*Myc* cell injection, previous experiments have shown statistical significance starting from six mice per group. Survival was determined as the time between the intravenous injection of the cells and the time when mice had to be euthanized for ethical reasons (end point). End point was evaluated by blinding. Survival functions were estimated using the Kaplan-Meier method and compared with the log-rank test.

### RNA extraction, RNA-seq, and real-time qPCR

Total RNA was extracted from Eμ-*Myc* cells using the RNeasy mini kit (74104, Qiagen) according to the manufacturer’s instructions. Reverse transcription was performed using the Omniscript RT kit (205113, Qiagen).

RNA-seq was performed on Illumina Hiseq2000 at the IPMC functional genomics platform (France). Libraries were generated from 1 μg of total RNA using TruSeq Stranded mRNA kit (Illumina). Libraries were then quantified with Qubit dsDNA High Sensitivity Assay Kit (Invitrogen) and pooled. Four nanomolar of this pool was loaded on a NextSeq 500 High Output Flowcell and sequenced on a NextSeq 500 platform (Illumina) with 2  ×  75–base pair paired-end chemistry. Analysis of the obtained libraries sequences (reads) was performed with DESeq2-Bioconductor after assessing quality of the data and gene counts and normalization.

The relative mRNA levels of murine *Asns* were obtained by real-time qPCR on the StepOne Real-Time PCR System (Thermo Fisher Scientific) using the TaqMan assay primer set for murine *Asns* (Mm01137310_g1, Thermo Fisher Scientific) and the TaqMan Universal PCR Master Mix (4304437, Thermo Fisher Scientific) according to the manufacturer’s instructions. The relative mRNA levels of murine *Asns* were normalized to mouse *Rplp0* (Mm00725448_s1, Thermo Fisher Scientific).

### Generation of V5-tagged murine ASNS-overexpressing cells

Total RNA was extracted from Eμ-*Myc* cells, and reverse transcription was performed as previously described. The resulting cDNA was used to amplify by PCR murine *Asns* coding sequence tagged to V5 (mASNS-V5) using a recombinant *Taq* DNA polymerase (10342020, Thermo Fisher Scientific) according to the manufacturer’s instructions and the following primers: forward 5′-CGCGGATCCATGTGTGGCATCTGGGCCCTCTTCGGCAGC-3′ and reverse 5′-TTTTCCTTTTGCGGCCGCCTACGTAGAATCGAGACCGAGGAGAGGGTTAGGGATAGGCTTACCAGCTTTGGCAGCTGACTTATAATGGGTCAG-3′. The PCR product was cloned into the pCR 2.1-TOPO TA vector using the TOPO TA Cloning Kit for Subcloning (K455040, Thermo Fisher Scientific) according to the manufacturer’s instructions. The plasmid was amplified in One Shot TOP10 chemically competent *E. coli* (C404003, Thermo Fisher Scientific) with ampicillin (A9518, Sigma-Aldrich) selection, purified (740414.50, Macherey-Nagel), and sequenced to confirm the cloning of the *wild-type* murine *Asns* coding sequence. Once confirmed, m*Asns-v5* sequence was removed from the pCR 2.1-TOPO TA vector using the Bam HI (R3136S, New England Biolabs) and Not I (R3189S, New England Biolabs) restriction sites. Upon gel purification (Monarch DNA Gel Extraction Kit, T10205, New England Biolabs), the 5′ overhangs were filled [DNA Polymerase I, Large (Klenow) Fragment, M0210S, New England Biolabs], and the m*Asns-v5* sequence was cloned into the Hpa I (R0105S, New England Biolabs) restriction site of the retroviral GFP-encoded pMIG vector (9044, Addgene) previously dephosphorylated (antarctic phosphatase, M0289S, New England Biolabs). Generated plasmid was amplified in MAX Efficiency Stbl2 competent cells (10268019, Thermo Fisher Scientific) with ampicillin selection, purified, and sequenced.

Self-inactivating retroviruses were generated by transient transfection of 293T cells (#CRL-1573, ATCC) and tittered as previously described (*21*). Briefly, 8.6 μg of the GFP-encoded pMIG empty vector (CTL) or GFP-encoded pMIG-m*Asns-v5* vector (ASNS OE) was cotransfected with 3 μg of envelope plasmid phCMV-VSV-G and 8.6 μg of MLV-Gag-Pol using the classical calcium phosphate method. Fifteen hours after transfection, 293T cells were incubated in Opti-MEM reduced serum media (11058021, Thermo Fisher Scientific) for 32 hours. Cell supernatant was centrifuged 3000*g* at 4°C overnight to concentrate retroviral particles prior to titration. Eμ-*Myc* cells were then transduced with the resulting retroviruses using a multiplicity of infection of 10. Seventy-two hours after transduction, GFP^+^ cells were sorted (SH800S Cell Sorter, Sony).

### Western blot analysis

For protein extraction, Eμ-*Myc* cells were washed in phosphate-buffered saline (PBS) and lysed in Laemmli buffer. Frozen tissue samples were homogenized using a stainless steel tissue grinder (1292, BioSpec Products), and protein was extracted using the Laemmli lysis buffer. After protein quantification (23225, Pierce BCA Protein Assay kit, Thermo Fisher Scientific), 30 μg of whole-cell protein lysates was separated on 8 to 15% SDS polyacrylamide gels and transferred onto polyvinylidene difluoride membranes (Millipore). Membranes were then blotted with antibodies against ASNS (14681-1-AP, Proteintech, RRID: AB_2060119), ATF4 (11815, Cell Signaling Technology, RRID: AB_2616025), and ERK2 (sc-1647, Santa Cruz Biotechnology, RRID: AB_627547). Immunoreactive bands were detected with anti-mouse (7076, Cell Signaling Technology, RRID: AB_330924) or anti-rabbit (7074S, Cell Signaling Technology, RRID: AB_2099233) immunoglobulin G horseradish peroxidase–linked antibodies. Immunoblots were visualized (FUJIFILM LAS4000) by chemiluminescence using Pierce ECL Western Blotting substrates (32106, Pierce ECL, Thermo Fisher Scientific). When indicated, Western blot quantification was performed using ImageJ software.

Whole-cell lysate obtained from Eμ-*Myc* cells treated for 6 hours with tunicamycin (1 μg/ml) (T7765, Sigma-Aldrich) was used as a positive control for ATF4 expression.

### Cell proliferation assay

A total of 250,000 live Eμ-*Myc* cells/ml were seeded in DMEM-GlutaMAX media (31966047, Thermo Fisher Scientific) supplemented with 10% FBS, 2-mercaptoethanol (50 μM), Hepes pH 7.4 (10 mM), penicillin-streptomycin (100 U/ml), in the presence or absence of l-asparagine (0.37 mM), and incubated at 37°C, 5% CO_2_ for the indicated time. The number of live cells at each time point was determined by 4′,6-diamidino-2-phenylindole (DAPI) (0.5 μg/ml) staining and flow cytometry analysis (Miltenyi Biotec). The proliferation index was calculated dividing the number of live cells at each time point by the initial number of live cells seeded.

### Cell competition assay

GFP^−^ (nontransduced) and GFP^+^ (overexpressing murine ASNS-V5) Eμ-*Myc* cells were seeded together (ratio 1:1) at a concentration of 500,000 live cells/mL in DMEM high-glucose, no-glutamine media (11960085, Thermo Fisher Scientific) supplemented with 10% FBS, 2-mercaptoethanol (50 μM), Hepes pH 7.4 (10 mM), penicillin-streptomycin (100 U/ml), 1 mM sodium pyruvate (11360070, Thermo Fisher Scientific), 2 mM l-glutamine (25030081, Thermo Fisher Scientific), in the presence or absence of l-asparagine (0.37 mM), and incubated at 37°C, 5% CO_2_. Every 24 hours for 7 days, cells were labeled with DAPI (0.5 μg/ml) and immediately analyzed by flow cytometry (Miltenyi Biotec) to determine the percentages of GFP^−^ and GFP^+^ live (DAPI^−^) cells. Cells were diluted every 24 hours with fresh media to keep a concentration of 500,000 live cells/ml.

### Cell death assay

A total of 500,000 live Eμ-*Myc* cells/ml were seeded in DMEM high-glucose, no-glutamine media (11960085, Thermo Fisher Scientific) supplemented with 10% FBS, 2-mercaptoethanol (50 μM), Hepes pH 7.4 (10 mM), penicillin-streptomycin (100 U/ml), 1 mM sodium pyruvate (11360070, Thermo Fisher Scientific), 2 mM l-glutamine (25030081, Thermo Fisher Scientific), in the presence or absence of l-asparagine and/or ASNase [native or recombinant WT or mutant Q59L (*30*)] at indicated concentrations, and incubated at 37°C, 5% CO_2_ for the indicated time. Cells were then labeled with DAPI (0.5 μg/ml), and the percentage of dead cells (DAPI^+^ cells) was immediately analyzed by flow cytometry (Miltenyi Biotec). Data represent the average of the percentage of DAPI^+^ cells.

### Determination of glutamine and glutamate concentrations

ASNase (native, recombinant WT, or Q59L mutant) was incubated in DMEM high-glucose, no-glutamine media (#11960044, Thermo Fisher Scientific) supplemented with 10% FBS, 2-mercaptoethanol (50 μM), Hepes pH 7.4. (10 mM), l-glutamine (2 mM), sodium pyruvate (1 mM), and l-asparagine (0.37 mM) at 37°C, 5% CO_2_, in the presence or absence of Eμ-*Myc* cells for the indicated times (0, 3, 8, 24, and 48 hours). The concentrations of l-glutamine and l-glutamate (mM) in media (without cells) or in cultured cell supernatants were determined electroenzymatically using the YSI 2950 Biochemistry Analyzer (Yellow Springs Instruments).

### In vitro stable isotope tracing

A total of 500,000 live Eμ-*Myc* cells/ml were seeded in DMEM high-glucose, no-glutamine media (#11960044, Thermo Fisher Scientific) supplemented with 10% FBS, 2-mercaptoethanol (50 μM), Hepes pH 7.4 (10 mM), penicillin-streptomycin (100 U/ml), sodium pyruvate (1 mM), 2 mM [U-^13^C_5_]-l-glutamine (CLM-1822-H-0.5, Cambridge Isotope Laboratories) or 2 mM ^15^N(amide)-l-glutamine (NLM-557-1, Cambridge Isotope Laboratories), indicated concentrations of l-asparagine (0, 0.009, 0.09, and 0.37 mM), and in the presence or absence of ASNase (0.003 IU/ml) for 6 or 24 hours. At end point, Eμ-*Myc* cells were stained with DAPI (0.5 μg/ml) and immediately analyzed by flow cytometry to determine the number of live cells per milliliter. A total of 1 × 10^6^ live Eμ-*Myc* cells per replicate were collected, washed twice in prechilled PBS (400*g*, 5 min, 15°C centrifugation), and snap frozen. Samples were stored at −80°C for further LC-MS–based metabolomic analyses. Results have been corrected for the presence of naturally occurring ^13^C stable isotopes using Metabolite AutoPlotter (*50*).

### In vivo stable isotope tracing

Mouse primary Eμ-*Myc* lymphoma cells were transferred into syngeneic, nontransgenic, 6-week-old C57BL/6JOlaHsd females (Envigo) by intravenous injection of 100,000 live cells per recipient mouse (in 150 μl of sterile PBS). Upon lymphoma development (determined by inguinal lymph node palpation), mice received two consecutive discrete bolus of [U-^13^C_5_]-l-glutamine (0.3 mg/kg mouse weight in 150 μl of sterile NaCl 0.9%) of 20-min interval, by intraperitoneal injection. Blood was collected from the tail vein 40 min after the first [U-^13^C_5_]-glutamine injection, and plasma was isolated by centrifugation at 6000*g* for 2 min and snap frozen. Mice were then immediately euthanized by cervical dislocation; tumors were harvested and snap frozen. The same procedure was performed in 6-week-old WT C57BL/6JOlaHsd females (Envigo) not bearing Eμ-*Myc* lymphomas, from which the pancreas was harvested and snap frozen. All the collected samples were stored at −80°C for further LC-MS–based metabolomic analyses. Food was given ad libitum. No randomization was performed. Results have been corrected for the presence of naturally occurring ^13^C stable isotopes using Metabolite AutoPlotter (*50*).

### Targeted LC/MS metabolites analyses

For in vitro samples, frozen pellets of 1 × 10^6^ Eμ-Myc cells were used for metabolite extraction. For tissues, frozen samples were homogenized using a stainless steel tissue grinder (1292, BioSpec Products), and 10 mg of tissue was used for metabolite extraction. Extraction solution was composed of 50% methanol, 30% acetonitrile (ACN), and 20% water. The volume of the extraction solution was adjusted to the cell number or tissue weight (1 ml per 1 million cells or 1 ml per 50 mg of tissues, respectively). Plasma was diluted 20-fold with the same extraction solvent. After addition of extraction solution, samples were vortexed for 5 min at 4°C and centrifuged at 16,000*g* for 15 min at 4°C. The supernatants were collected and stored at −80°C until analysis. LC-MS analyses were conducted on a QExactive Plus Orbitrap mass spectrometer equipped with an Ion Max source and a HESI II probe coupled to a Dionex UltiMate 3000 UPLC system (Thermo Fisher Scientific). External mass calibration was performed using a standard calibration mixture every 7 days, as recommended by the manufacturer. The 5-μl samples were injected onto a ZIC-pHILIC column [150 mm × 2.1 mm; internal diameter (i.d.), 5 μm] with a guard column (20 mm × 2.1 mm; i.d., 5 μm) (Millipore) for LC separation. Buffer A was 20 mM ammonium carbonate, 0.1% ammonium hydroxide (pH 9.2), and buffer B was ACN. The chromatographic gradient was run at a flow rate of 0.200 μl min^−1^ as follows: 0 to 20 min, linear gradient from 80 to 20% of buffer B; 20 to 20.5 min, linear gradient from 20 to 80% of buffer B; 20.5 to 28 min, 80% buffer B. The mass spectrometer was operated in full scan, polarity switching mode with the spray voltage was set to 2.5 kV, and the heated capillary was held at 320°C. The sheath gas flow was set to 20 U, the auxiliary gas flow to 5 U, and the sweep gas flow to 0 U. The metabolites were detected across a mass range of 75 to 1000 mass/charge ratio (*m*/*z*) at a resolution of 35,000 (at 200 *m*/*z*) with the automatic gain control target at 10^6^ and the maximum injection time at 250 ms. Lock masses were used to ensure mass accuracy below 5 parts per million. Data were acquired with Thermo Xcalibur software (Thermo Fisher Scientific). The peak areas of metabolites and isotopologues were determined using Thermo TraceFinder software (Thermo Fisher Scientific), identified by the exact mass of each singly charged ion and by the known retention time on the high-performance LC column. For the absolute quantification, standard addition method was used (*51*).

### Immunohistochemistry

Sections (4 μm) of formalin-fixed, paraffin-embedded DLBCL biopsies [training cohort (*21*)] were treated for deparaffinization, rehydration, and antigen retrieval using standard procedures (EnVision FLEX reagents, Agilent, Dako). Antibodies against CD79A (M7050, Agilent, Dako, RRID: AB_2244527), CD20 (M0755, Agilent, Dako, RRID: AB_2282030), and ASNS (HPA029318, Sigma-Aldrich, RRID: AB_10602389) were used for immunostainings performed on an automated system (Autostainer link 48, Dako) as previously described (*21*). Antigen retrieval was performed at specific pH prior to incubation with the indicated antibodies: pH 6.0 prior anti-CD79A, anti-CD20, and pH 9.0 prior anti-ASNS. Primary antibodies were diluted in EnVision FLEX antibody diluent (anti-CD20 is prediluted; anti-CD79A, dilution 1:100; anti-ASNS, dilution 1:100).

### Other statistical analysis

Comparative tests were considered significant if two-sided *P* < 0.05. For ex vivo and in vitro experiments, results are expressed as means ± SD, and statistical differences were determined by two-tailed Student’s *t* test or two-way analysis of variance (ANOVA) test. A *P* < 0.05 was considered to indicate statistical significance (*P < 0.05; **P < 0.01; ***P < 0.001; ****P < 0.0001; ns, not significant). All statistical analyses were performed using GraphPad Prism 9 software.

## Notes

View/request a protocol for this paper from *Bio-protocol*.
