# Supplementary material for: Tumoral microenvironment prevents de novo asparagine biosynthesis in B cell lymphoma, regardless of ASNS expression
Source: Sci Adv. 2022 Jul 6;8(27):eabn6491. doi: 10.1126/sciadv.abn6491 (PMC9258813; doi:10.1126/sciadv.abn6491)
Supplement: Supplementary file 1 — Figs. S1 to S5 Table S1 [file sciadv.abn6491_sm.pdf]

Science Advances
AAAS

Supplementary Materials for

Tumoral microenvironment prevents de novo asparagine biosynthesis in B cell lymphoma, regardless of ASNS expression

Manuel Grima-Reyes *et al.*

Corresponding author: Johanna Chiche, chiche@unice.fr; Jean-Ehrland Ricci, ricci@unice.fr

Sci. Adv. **8**, eabn6491 (2022)
DOI: 10.1126/sciadv.abn6491

This PDF file includes:

Figs. S1 to S5
Table S1

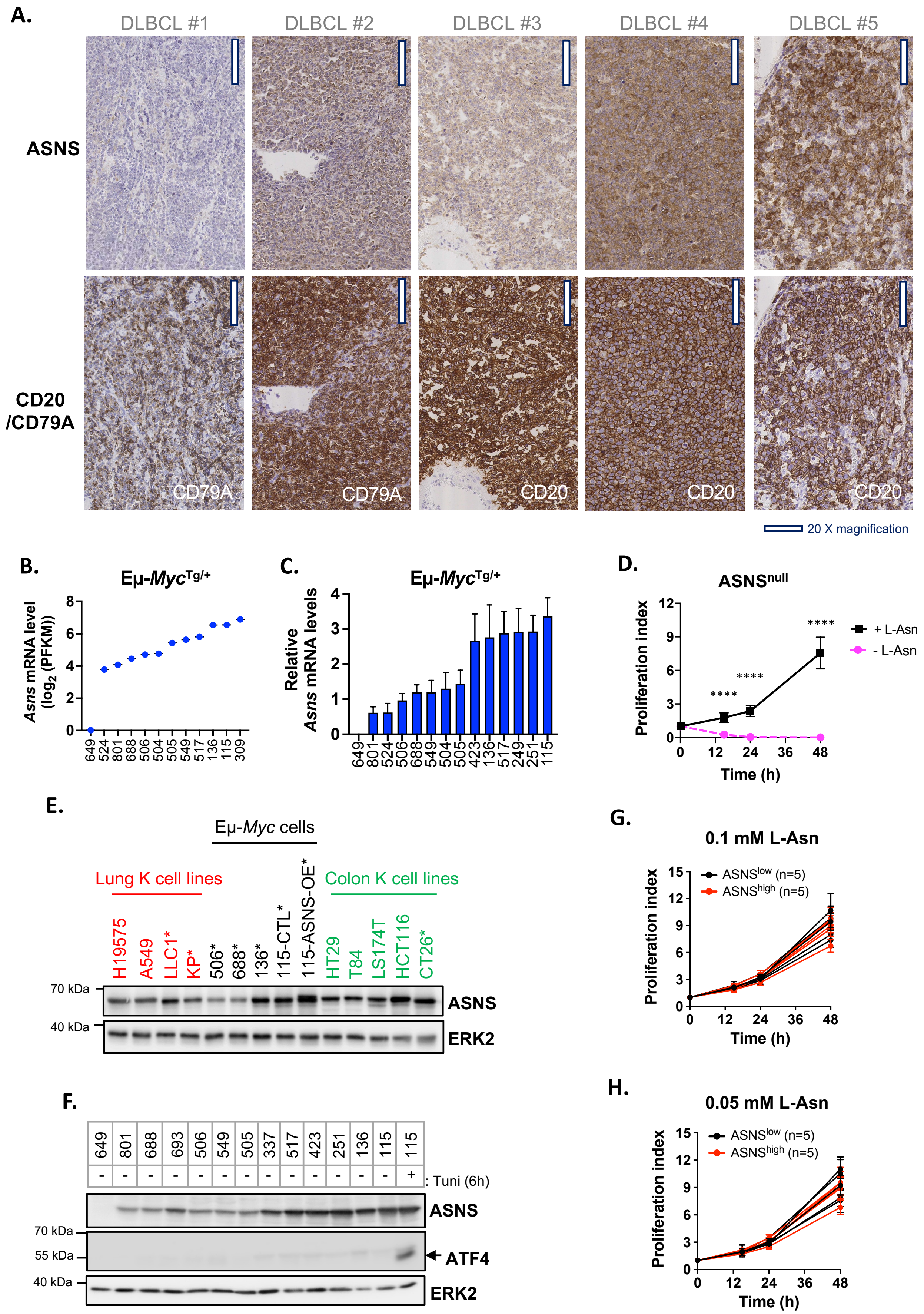
A.
DLBCL #1
DLBCL #2
DLBCL #3
DLBCL #4
DLBCL #5
ASNS
CD20
/CD79A
CD79A
CD79A
CD20
CD20
CD20
20 X magnification
B.
Eμ-Myc Tg/+
Asns mRNA level (log2 (PFKM))
C.
Eμ-Myc Tg/+
Relative Asns mRNA levels
D.
ASNS null
Proliferation index
+ L-Asn
- L-Asn
Time (h)
E.
Eμ-Myc cells
Lung K cell lines
Colon K cell lines
H19575
A549
LLC1*
KP*
506*
688*
136*
115-CTL*
115-ASNS-OE*
HT29
T84
LS174T
HCT116
CT26*
70 kDa
40 kDa
ASNS
ERK2
F.
: Tuni (6h)
70 kDa
55 kDa
40 kDa
ASNS
ATF4
ERK2
G.
0.1 mM L-Asn
ASNS low (n=5)
ASNS high (n=5)
Proliferation index
Time (h)
H.
0.05 mM L-Asn
ASNS low (n=5)
ASNS high (n=5)
Proliferation index
Time (h)

Figure S1., related to Figure 1

A. Serial sections of formalin-fixed paraffin-embedded DLBCL biopsies from five patients were immunostained for ASNS and CD20 or CD79A. The white scale bar represents 100 µm (20 X magnification).

B. *Asns* mRNA levels determined by RNA-sequencing in primary Eµ-*Myc* cells isolated from B-cell lymphomas of 12 independent Eµ-$Myc^{Tg/+}$ transgenic mice. Data are expressed as base 2 logarithm of *Asns* fragments per kilobase million (FPKM).

C. Relative *Asns* mRNA levels determined by real-time qPCR (normalized by *Rplp0)* in primary Eµ-*Myc* cells isolated from 14 independent Eµ-$Myc^{Tg/+}$ transgenic mice. Data are expressed as mean ± SD of n=3 independent experiments.

D. Proliferation index of primary Eµ-*Myc*-$ASNS^{null}$ cells (isolated from the Eµ-$Myc^{Tg/+}$mouse #649) in the presence (+ L-Asn) or absence (-L-Asn) of L-asparagine (0.37 mM). At each time point (0, 15h, 24h and 48h), cells were stained with DAPI and analyzed by flow cytometry to determine the number of live (DAPI negative) cells per mL. Data are expressed as mean ± SD of n=3 independent experiments.

E. Immunoblot for the detection of ASNS protein in whole-cell lysates prepared from mouse primary Eµ-*Myc* cells ($ASNS^{low}$ and $ASNS^{high}$), mouse and human lung (H1975, A549, LLC1, $KRAS^{G12D}$-p53-/- referred as KP) and colon (HT29, T84, LS174Tr, HCT116, CT26) carcinoma cell lines incubated in the presence of L-asparagine (0,37 mM) for 24h. ERK2 was used as a loading control. Murine cell lines are annotated with a star.

F. Whole-cell lysates prepared from Eµ-*Myc* cells were analyzed by immunoblot for the detection of indicated proteins. Each lane corresponds to a B-cell lymphoma harvested from a Eµ-$Myc^{Tg/+}$ transgenic mouse (n=13 independent mice). Eµ-*Myc*-$ASNS^{high}$ cells (isolated from the Eµ-$Myc^{Tg/+}$ mouse #115) were incubated in the presence (+) or absence (-) of tunicamycin (1 µg/mL) for 6h as a positive control for ATF4 expression. ERK2 was assessed in all samples as a loading control.

G. H. Proliferation index of primary Eµ-*Myc* cells isolated from 10 independent Eµ-$Myc^{Tg/+}$ mice, according to ASNS expression levels (low, n=5 or high, n=5). Cells were cultivated in the presence of 0.1 mM (**G.**) or 0.05 mM of L-asparagine (**H.**) At each time point (0, 15h, 24h and 48h), the number of live cells per mL was determined by DAPI staining through flow cytometry. Data are expressed as mean ± SD of n=3 independent experiments.

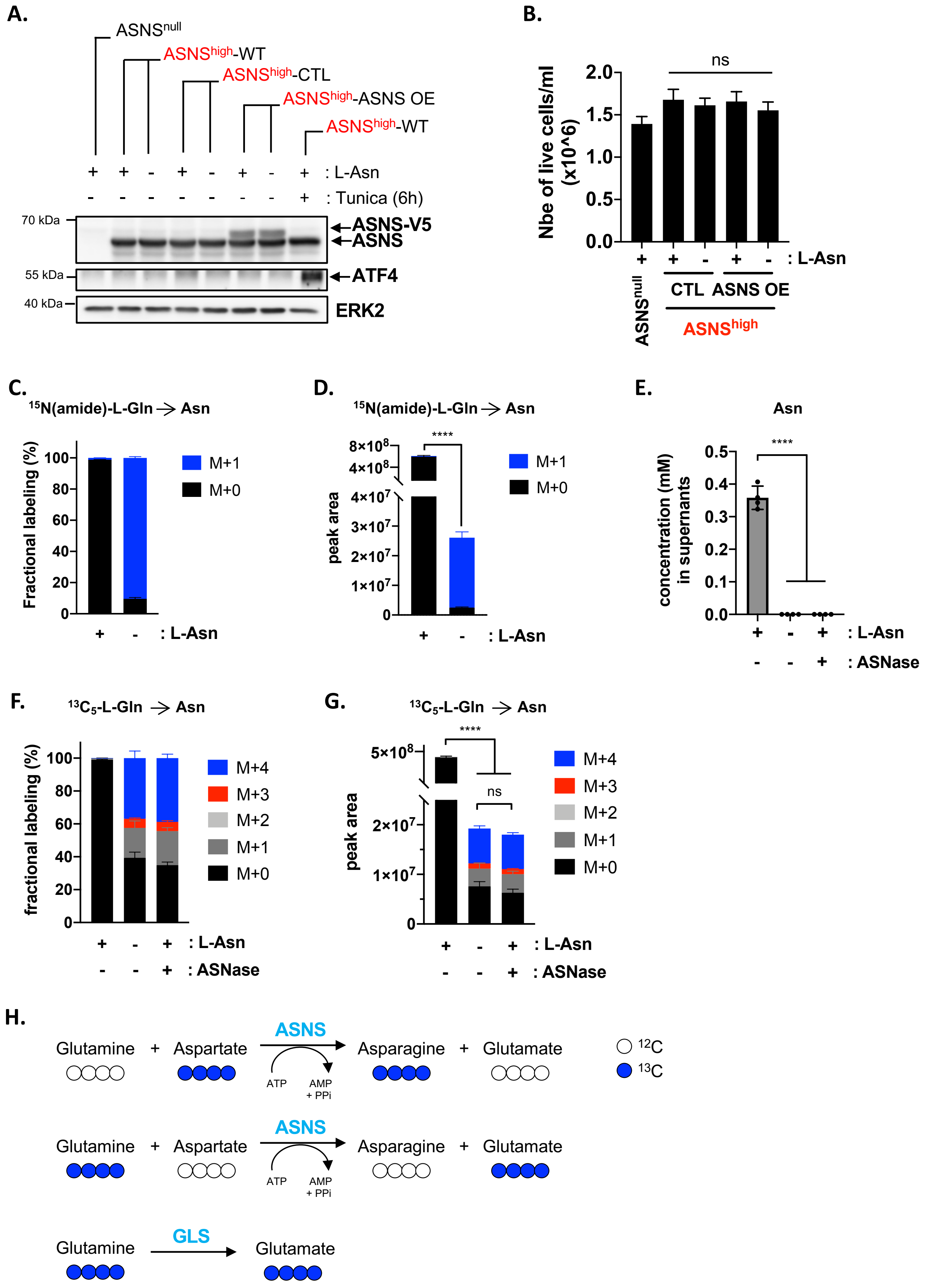

A.
ASNSnull
ASNShigh-WT
ASNShigh-CTL
ASNShigh-ASNS OE
ASNShigh-WT
: L-Asn
: Tunica (6h)
70 kDa
55 kDa
40 kDa
ASNS-V5
ASNS
ATF4
ERK2
B.
Nbe of live cells/ml (x10^6)
ns
: L-Asn
ASNSnull
CTL
ASNS OE
ASNShigh
C.
15N(amide)-L-Gln → Asn
Fractional labeling (%)
M+1
M+0
: L-Asn
D.
15N(amide)-L-Gln → Asn
peak area
****
M+1
M+0
: L-Asn
E.
Asn
concentration (mM) in supernants
****
: L-Asn
: ASNase
F.
13C5-L-Gln → Asn
fractional labeling (%)
M+4
M+3
M+2
M+1
M+0
: L-Asn
: ASNase
G.
13C5-L-Gln → Asn
peak area
****
ns
M+4
M+3
M+2
M+1
M+0
: L-Asn
: ASNase
H.
Glutamine + Aspartate
ASNS
ATP
AMP + PPi
Asparagine + Glutamate
12C
13C
Glutamine + Aspartate
ASNS
Asparagine + Glutamate
Glutamine
GLS
Glutamate

Figure S2., related to Figure 1.

A. Eμ-*Myc*-ASNS^{null} (isolated from the Eμ-*Myc*$^{Tg/+}$mouse #649), wild-type (WT) Eμ-*Myc*-ASNS^{high} (isolated from the Eμ-*Myc*$^{Tg/+}$mouse #115) and Eμ-*Myc*-ASNS^{high} (#115) stably overexpressing V5-tagged murine ASNS (ASNS^{high}-ASNS OE) or control vector (ASNS^{high}-CTL) were incubated in the presence (+) or absence (-) of L-asparagine (0.37 mM) for 24h. Corresponding whole-cell lysates were immunoblotted with the indicated antibodies. Tunicamycin treatment (1 μg/mL) for 6h was used as a positive control for ATF4 expression. ERK2 was used as a loading control.

B. The number of live (DAPI negative) Eμ-*Myc*-ASNS^{null} cells (isolated from the Eμ-*Myc*$^{Tg/+}$mouse #649) and of live Eμ-*Myc*-ASNS^{high} cells (isolated from the Eμ-*Myc*$^{Tg/+}$mice #115) stably overexpressing control vector (CTL) or V5-tagged murine ASNS (ASNS OE) incubated for 24h in DMEM high glucose no glutamine media supplemented with 2 mM of [U-$^{13}C_5$]-L-glutamine, 1 mM of sodium pyruvate, with (+) or without (-) L-asparagine (as in Figure 1F-H) was determined by flow cytometry.

C-D. Fractional labeling (**C.**) and peak area (**D.**) of asparagine ^{13}C isotopologues in primary Eμ-*Myc*-ASNS^{high} cells (isolated from the Eμ-*Myc*$^{Tg/+}$mouse #115) as in Figures 1G-H with 2 mM of ^{15}N(amide)-L-glutamine. Data are expressed as mean ± SD of n=4 biological replicates. Results have been corrected for the presence of naturally occurring ^{13}C stable isotopes using Metabolite AutoPlotter. For **D.** p-value was determined by t-test; ****, $p < 0.0001$.

E. Concentration of L-asparagine in the supernatant of Eμ-*Myc*-ASNS^{high} cells treated for 24h with 0.003 IU/ml of ASNase. Data are expressed as mean ± SD of n=4 biological replicates. P-value was from t-test; ****, $p < 0.0001$.

F. G. Fractional labeling (**F.**) and peak area (**G.**) of asparagine ^{13}C isotopologues in primary Eμ-*Myc*-ASNS^{high} cells (isolated from the Eμ-*Myc*$^{Tg/+}$mouse #115) incubated in DMEM high glucose no glutamine media supplemented with 2 mM of [U-$^{13}C_5$]-L-glutamine, 1 mM of sodium pyruvate, 0.37 mM of L-asparagine and in the presence (+) or absence (-) of 0.003 IU/ml of ASNase for 24h. Data are expressed as mean ± SD of n=4 biological replicates. Results have been corrected for the presence of naturally occurring ^{13}C stable isotopes using Metabolite AutoPlotter. For **G.** p-value was determined by t-test; ****, $p < 0.0001$.

H. Schema depicting reactions metabolizing glutamine, catalyzed by ASNS or GLS. Blue circles represent labeled carbons from [U-$^{13}C_5$]-L-glutamine.

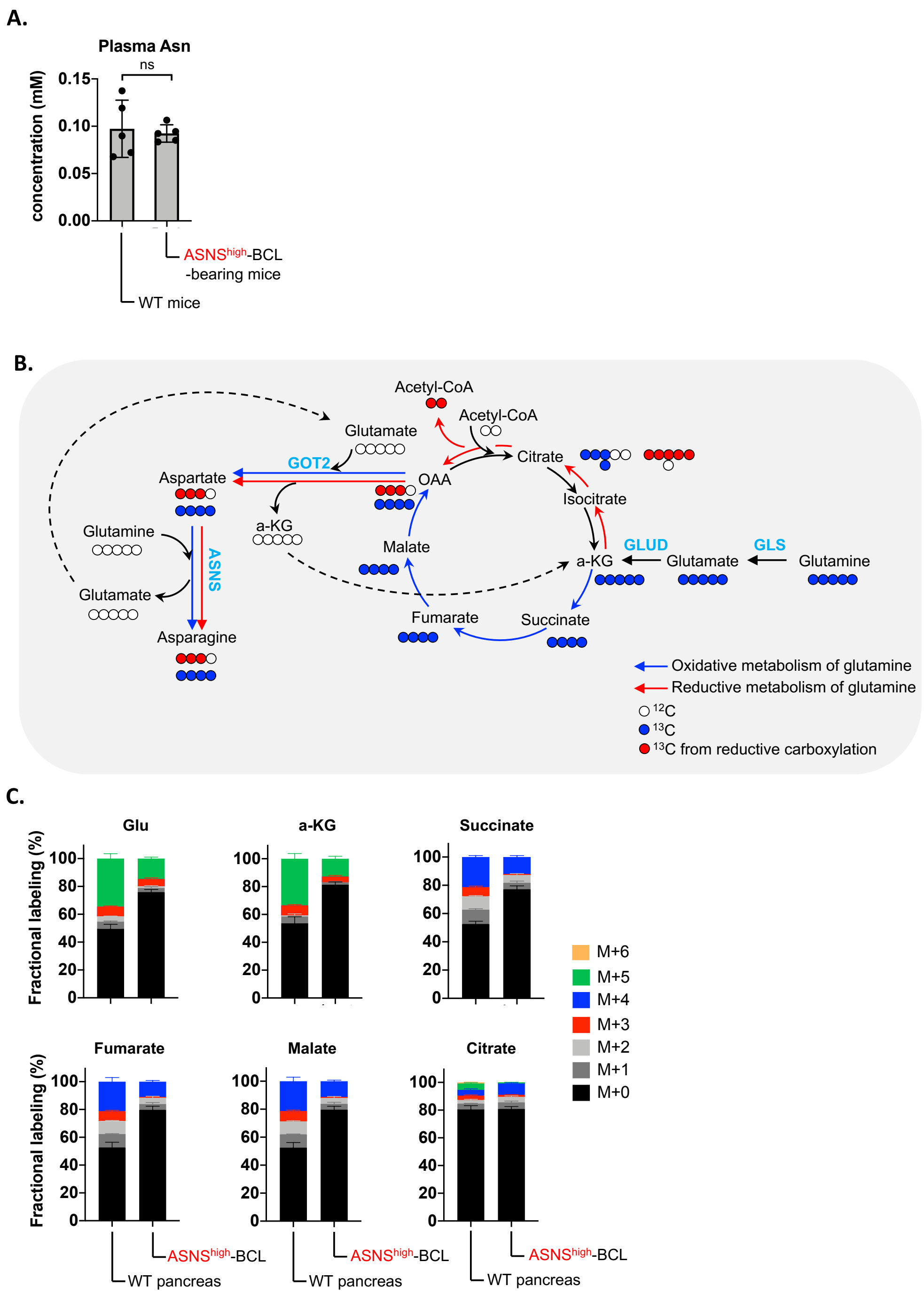

Figure S3., related to Figure 2.

A. Concentration of asparagine in the plasma of healthy wild-type C57Bl/6 mice (n=5) and of Eμ-*Myc*-ASNS^{high}-BCL-bearing mice (n=5) obtained as described in Figure 2C. Data are expressed as mean ± SD (n=5 mice/group). P-value was determined by t-test; ns, not significant.

B. Schematic depicting oxidative and reductive metabolism of glutamine from [U-$^{13}C_5$]-L-glutamine.

C. Fractional labeling of glutamate and TCA cycle intermediate ^{13}C isotopologues in the pancreas of healthy C57Bl/6 mice (n=5) and in axillary ASNS^{high}-BCL of ASNS^{high}-BCL-bearing C57Bl/6 mice (n=5), following two discrete boluses of [U-$^{13}C_5$]-L-glutamine in mice, as described in Figure 2C.

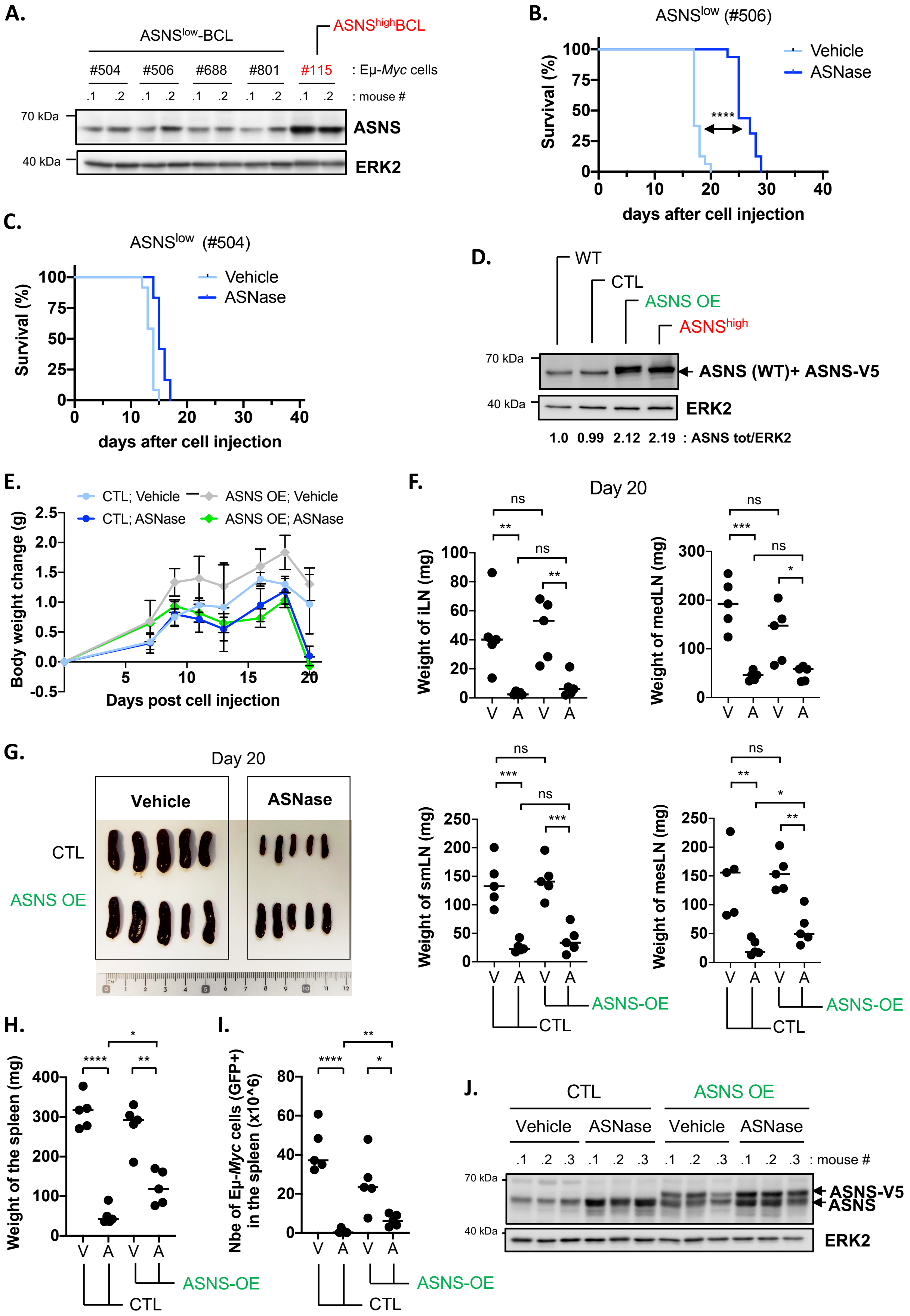
A.
ASNSlow-BCL
ASNShighBCL
#504 #506 #688 #801 #115 : Eµ-Myc cells
.1 .2 .1 .2 .1 .2 .1 .2 .1 .2 : mouse #
70 kDa
ASNS
40 kDa
ERK2
B.
ASNSlow (#506)
Survival (%)
Vehicle
ASNase
****
days after cell injection
C.
ASNSlow (#504)
Survival (%)
Vehicle
ASNase
days after cell injection
D.
WT
CTL
ASNS OE
ASNShigh
70 kDa
ASNS (WT)+ ASNS-V5
40 kDa
ERK2
1.0 0.99 2.12 2.19 : ASNS tot/ERK2
E.
CTL; Vehicle
ASNS OE; Vehicle
CTL; ASNase
ASNS OE; ASNase
Body weight change (g)
Days post cell injection
F.
Day 20
Weight of iLN (mg)
Weight of medLN (mg)
Weight of smLN (mg)
Weight of mesLN (mg)
ns
V A V A
ASNS-OE
CTL
G.
Day 20
Vehicle
ASNase
CTL
ASNS OE
H.
Weight of the spleen (mg)
V A V A
ASNS-OE
CTL
I.
Nbe of Eµ-Myc cells (GFP+) in the spleen (x10^6)
V A V A
ASNS-OE
CTL
J.
CTL
ASNS OE
Vehicle ASNase Vehicle ASNase
.1 .2 .3 .1 .2 .3 .1 .2 .3 .1 .2 .3 : mouse #
70 kDa
ASNS-V5
ASNS
40 kDa
ERK2

Figure S4., related to Figure 3.

A. Whole-cell lysates prepared from axillary BCL harvested from syngeneic C57Bl/6 mice intravenously injected with distinct primary Eµ-*Myc*-ASNS^{low} cells (isolated from Eµ-*Myc*$^{Tg/+}$ mice #504, #506, #688 and #801) or with Eµ-*Myc*-ASNS^{high} cells (isolated from the Eµ-*Myc*$^{Tg/+}$ mouse #115) were analyzed by immunoblot for ASNS expression (n=2 mice/group). ERK2 was used as a loading control.

B. Kaplan-Meier curves for the survival of syngeneic wild-type C57Bl/6 mice intravenously injected with primary Eµ-*Myc*-ASNS^{low} cells (isolated from the Eµ-*Myc*$^{Tg/+}$ mouse #506) and treated 7 days later with Vehicle (NaCl 0.9%) or ASNase (2500 IU/kg) every two days, three times a week (n=16 mice/group). P-value was determined by log-rank test; ****, $p < 0.0001$.

C. As in **B.** with primary Eµ-*Myc*-ASNS^{low} cells isolated from the Eµ-*Myc*$^{Tg/+}$ mouse #504 (n=12 mice/group).

D. As in figure **3C.** using a 15% acrylamide gel.

E. Average body weight loss of syngeneic wild-type C57Bl/6 mice intravenously injected with control-(CTL) and V5-tagged murine ASNS-(ASNS OE) overexpressing Eµ-*Myc*-ASNS^{low} cells as described in Figure 3E. On day 7, mice received intraperitoneal injections of Vehicle (NaCl 0.9%) or ASNase (2500 IU/kg) every two days, three times a week for a total of six injections. Mice had access to food and water *ad libitum*. Data are presented as mean ± SEM (n=5 mice/group). P-value was determined by t-test; *, $p < 0.05$; **, $p < 0.01$; ***, $p < 0.001$; ns, not significant.

F. Weight of inguinal (iLN), sub-maxillary (smLN), mediastinal (medLN) and mesenteric (mesLN) lymph nodes harvested on day 20 from mice described in **E.** and in Figure 3E. (n=5 mice/group). P-value was determined by t-test; *, $p < 0.05$; **, $p < 0.01$; ***, $p < 0.001$; ns, not significant.

G. H. Size (**G.**) and weight (**H.**) of the spleen harvested on day 20 from mice described in **E.** and in Figure 3E (n=5 mice/group). P-value was determined by t-test; *, $p < 0.05$; **, $p < 0.01$; ***, $p < 0.001$.

I. The number of live (DAPI negative) Eµ-*Myc* cells (GFP positive) in the mice spleen illustrated in **G.** was determined by flow cytometry (n=5 mice/group). P-value was determined by t-test; *, $p < 0.05$; **, $p < 0.01$; ****, $p < 0.0001$; ns, not significant.

J. Western blot analysis of ASNS expression in three representative axillary BCL harvested at end point from mice presented in Figure 3H. (from n=3 mice/group). ERK2 was assessed in all samples as a loading control.

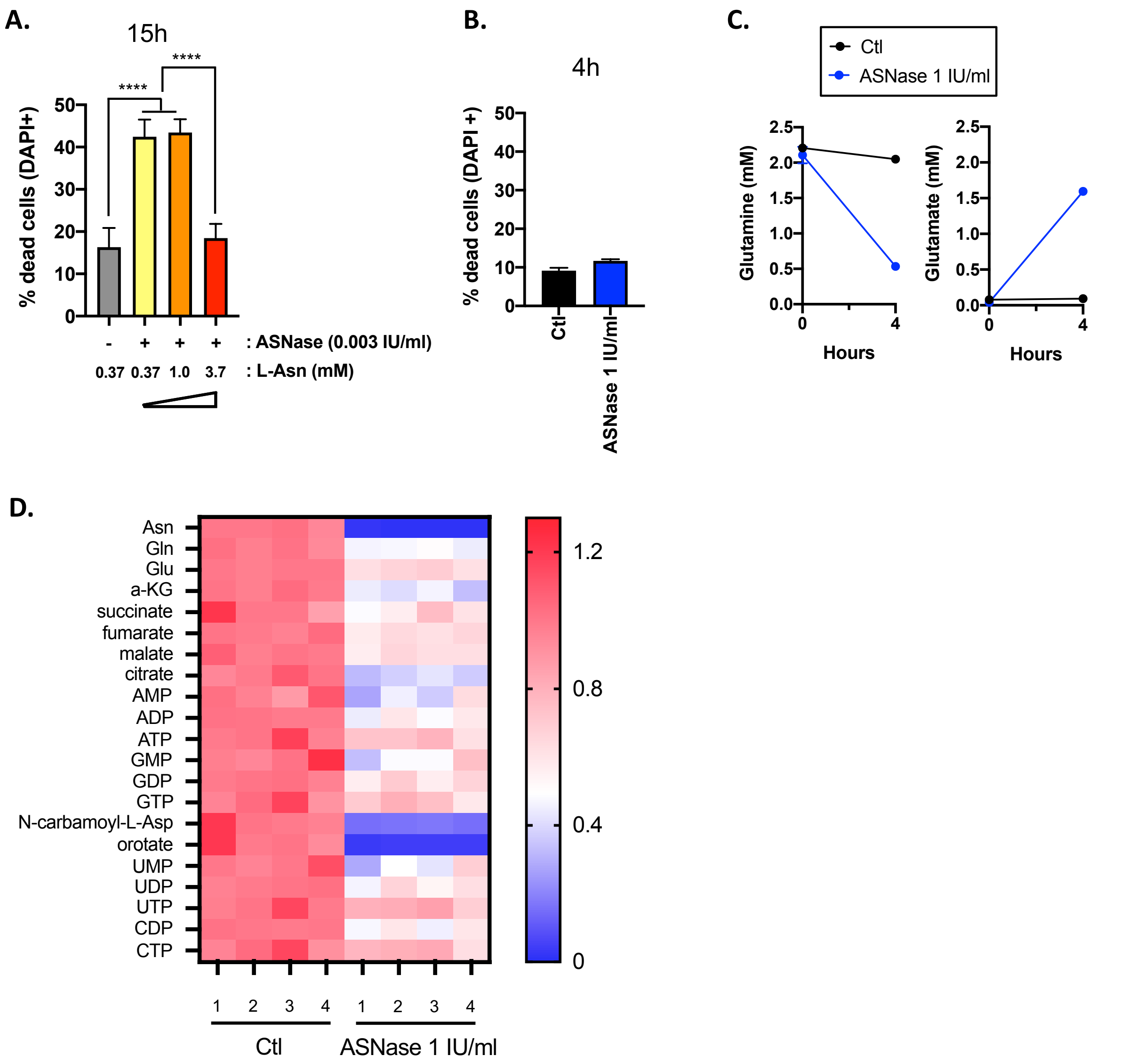

Figure S5., related to Figure 4.

A. Eμ-*Myc*-ASNS^{low} cells (isolated from Eμ-*Myc*$^{Tg/+}$ mouse #506) were incubated in DMEM high glucose no glutamine media supplemented with L-glutamine (2 mM), sodium pyruvate (1 mM) and L-asparagine (at indicated concentrations), in the presence or absence (-) of 0.003 U/ml of ASNase for 15h. The percentage of dead cells was determined by DAPI staining and analyzed by flow cytometry. Data are expressed as mean ± SD (n=3 independent experiments) P-value was determined by t-test; ****, $p < 0.0001$.

B. Eμ-*Myc*-ASNS^{low} cells (isolated from Eμ-*Myc*$^{Tg/+}$ mouse #506) were incubated in DMEM high glucose no glutamine media supplemented with L-glutamine (2 mM), sodium pyruvate (1 mM) and L-asparagine (0.37 mM), in the presence or absence (Ctl) of ASNase 1 IU/ml for 4 hours. The percentage of dead cells was determined by DAPI staining and analyzed by flow cytometry. Data are expressed as mean ± SD (n=3 independent experiments).

C. Glutamine and glutamate concentration in the supernatant of cells presented in **B.**

D. Heatmap representation of the relative abundance of TCA cycle metabolites and precursors of nucleotides in Eμ-*Myc*-ASNS^{low} cells (isolated from Eμ-*Myc*$^{Tg/+}$ mouse #506) incubated in DMEM high glucose no glutamine media supplemented with L-glutamine (2 mM), sodium pyruvate (1 mM) and L-asparagine (0.37 mM), in the presence or absence (Ctl) of ASNase 1 U/ml for 4 hours, as described in **B.** n=4 biological replicates/group.

From *(23)*				From *(24)*			
	Asns				***Asns***		
	**High (n = 116)**	**Low (n = 117)**	**P-value**		**High (n = 307)**	**Low (n = 307)**	**P-value**
**Mean, age ± SD (y)**	60.55 ± 15.7	59.77 ± 16.7	0.71	**Mean, age ± SD (y)**	60.8 ± 14.9	60.05 ± 16.6	0.56
**Gender**			1	**Gender**			0.94
Male	67 (57.76%)	67 (57.26%)		Male	176 (57.33%)	174 (56.68%)	
Female	49 (42.24%)	50 (42.74%)		Female	131 (42.67%)	133 (43.32%)	
**Ann Arbor Stage (226/233)**			0.14	**Ann Arbor Stage (603/614)**			0.13
> 2	54 (46.55%)	67 (57.26%)		> 2	172 (56.03%)	192 (62.54%)	
≤ 2	58 (50.00%)	47 (40.17%)		≤ 2	129 (42.02%)	110 (35.83%)	
**Extranodal sites (203/233)**			0.53	**Extranodal sites (580/614)**			0.92
≥ 2	17 (14.66%)	13 (11.11%)		≥ 2	67 (21.82%)	70 (22.80%)	
< 2	84 (72.41%)	89 (76.07%)		< 2	221 (71.99%)	222 (72.31%)	
**ECOG performance status (210/233)**			0.23	**ECOG performance status (576/614)**			1
> 1	31 (18.10%)	21 (17.95%)		> 1	77 (25.08%)	77 (25.08%)	
≤ 1	77 (66.38%)	81 (69.23%)		≤ 1	210 (68.40%)	212 (69.06%)	
**LDH (192/233)**			0.25	**LDH (561/614)**			0.15
> ULN	51 (43.97%)	42 (35.90%)		> ULN	166 (54.07%)	144 (46.91%)	
≤ ULN	45 (38.79%)	54 (46.15%)		≤ ULN	118 (38.44%)	133 (43.32%)	
**IPI (178/233)**			0.10	**IPI (514/614)**			0.62
0-1	33 (28.45%)	37 (31.62%)		0-1	84 (27.36%)	78 (25.41%)	
2-3	40 (34.48%)	44 (37.61%)		2-3	114 (37.13%)	126 (41.04%)	
4-5	17 (14.66%)	7 (5.98%)		4-5	53 (17.26%)	49 (15.96%)	

Table S1: Univariate analysis of *Asns* transcripts expression with biological and clinical prognostic factors associated with tumor burden and disease progression in DLBCL patient samples, from two publicly available datasets.
